# Usefulness of low tidal volume ventilation strategy for patients with acute respiratory distress syndrome: a systematic review and meta-analysis

**DOI:** 10.1038/s41598-022-13224-y

**Published:** 2022-06-04

**Authors:** Ryohei Yamamoto, Satoru Robert Okazaki, Yoshihito Fujita, Nozomu Seki, Yoshufumi Kokei, Shusuke Sekine, Soichiro Wada, Yasuhiro Norisue, Chihiro Narita

**Affiliations:** 1 Department of Healthcare Epidemiology, School of Public Health in the Graduate School of Medicine, Kyoto University, Kyoto, Japan; 2 Department of Intensive Care Medicine, Kameda Medical Center, 929 Higashi-cho, Kamogawa, Chiba Japan; 3 Department of Anesthesiology and Intensive Care Medicine, Aichi Medical University, 1-1 Karimata, Yazako, Nagakute Japan; 4 Emergency Department, Toyama University Hospital, 2630, Sugitani, Toyama-shi, Toyama Japan; 5 Department of Emergency Medicine Trauma and Resuscitation Center, Tokyo Metropolitan Tama Medical Center, 2-8-29, Musashidai, Fuchu, Tokyo Japan; 6 Department of Anesthesiology, Tokyo Medical University, 6-7-1 Nishishinjyuku, Shinjuku-ku, Tokyo 160-0023 Japan; 7 Department of Pediatrics, Teine Keijinkai Hospital, 1-40, Maeda, Teine-ku, Sapporo, Hokkaido Japan; 8 Department of Emergency and Critical Care Medicine, Tokyo Bay Urayasu Ichikawa Medical Center, 3-4-32, Todaijima, Urayasu, Chiba Japan; 9 Departmenet of Emergency Medicine, Shizuoka General Hospital, 4-27-1, Kitaando, Aoiku, Shizuoka Japan

**Keywords:** Respiratory distress syndrome, Translational research

## Abstract

The effects of lower tidal volume ventilation (LTV) were controversial for patients with acute respiratory distress syndrome (ARDS). This systematic review and meta-analysis aimed to evaluate the use of LTV strategy in patients with ARDS. We performed a literature search on MEDLINE, CENTRAL, EMBASE, CINAHL, “Igaku-Chuo-Zasshi”, clinical trial registration sites, and the reference of recent guidelines. We included randomized controlled trials (RCTs) to compare the LTV strategy with the higher tidal volume ventilation (HTV) strategy in patients with ARDS. Two authors independently evaluated the eligibility of studies and extracted the data. The primary outcomes were 28-day mortality. We used the GRADE methodology to assess the certainty of evidence. Among the 19,864 records screened, 13 RCTs that recruited 1874 patients were included in our meta-analysis. When comparing LTV (4–8 ml/kg) versus HTV (> 8 ml/kg), the pooled risk ratio for 28-day mortality was 0.79 (11 studies, 95% confidence interval [CI] 0.66–0.94, I^2^ = 43%, n = 1795, moderate certainty of evidence). Subgroup-analysis by combined high positive end-expiratory pressure with LTV showed interaction (P = 0.01). Our study indicated that ventilation with LTV was associated with reduced risk of mortality in patients with ARDS when compared with HTV.

Trial registration: UMIN-CTR (UMIN000041071).

## Introduction

Acute respiratory distress syndrome (ARDS) is a life-threatening condition due to respiratory failure, often requiring mechanical ventilation for survival^1^. One of the most important aspects of ventilation management is minimizing pressure-related damage (barotrauma), capacity damage (volutrauma), and ventilator-induced lung injury (VILI)^2–4^.

Limiting the tidal volume is one of the strategies of lung protection that help in reducing adverse events due to mechanical ventilation^4,5^. Limiting the tidal volume results in lower levels of systemic inflammatory mediators^6^ and might prevent VILI by minimizing pressure-related and capacity damage^7–9^. On the contrary, lowering the tidal volume might also cause lung damage due to atelectasis, hypoxia, hypercapnia, patient discomfort, increased use of sedation, and cyclic atelectasis^10^.

Several randomized controlled trials (RCTs) that have analyzed the usefulness of lowering the tidal volume have shown inconsistent results^11–16^. The Cochrane Systematic Review of six trials that included 1297 patients with ARDS showed that 28-day mortality was significantly reduced by lung-protective ventilation, with a risk ratio (RR) of 0.74 (95% confidence interval [CI] 0.61–0.88)^5^. A recent systematic review of seven RCTs that included 1481 patients with ARDS demonstrated a trend towards lower risk of mortality, but the difference was insignificant (RR 0.87; 95% CI 0.70–1.08)^17^.

Lower tidal volume ventilation (LTV) has potentially relevant benefits; however, the certainty of evidence is imprecise. To develop the Japanese ARDS guidelines 2021, an updated systematic review is warranted. Therefore, this systematic review and meta-analysis aimed to evaluate the usefulness of the lower tidal volume ventilation strategy for patients with ARDS.

## Methods

### Protocol and registration

Our study adheres to the Preferred Reporting Items for Systematic Reviews and Meta-Analyses (PRISMA) protocol for RCTs^18^. The review protocol was submitted to the University Hospital Medical Information Network Clinical Trial Registry (UMIN-CTR) on July 7, 2020, before data extraction was initiated (identifier: UMIN000041071).

### Eligibility criteria

We included RCTs or cluster RCTs and excluded crossover trials, quasi-randomized, and non-randomized trials. The target population was intubated patients with ARDS (age ≥ 16 years). ARDS was defined according to the 1988 definition^19^, or the American-European Consensus Conference criteria^20^, or the Berlin definition^21^, or other authors’ definitions. We included studies that compared the LTV strategy with usual or higher tidal volume ventilation (HTV) strategy. We included a variety of tidal volume settings. For example, if there was a difference in the tidal volume between the two groups 24–72 h after the intervention due to differences in the method of setting tidal volume (specifying target tidal volume, a setting of the driving pressure, any protocol, or programmatic algorithms), we included them in this review (Additional File 1).

### Data sources and searches

We performed a literature search on MEDLINE through PubMed, EMBASE, Cochrane Central Register of Controlled Trials (CENTRAL), Cumulative Index to Nursing and Allied Health Literature (CINAHL), and Igaku-Chuo-Zasshi (Ichu-shi), a Japanese bibliographic database, from inception until July 2020. Our search strategies are described in an additional file (Additional File 1). We also performed searches for ongoing trials in the following trial registries: The World Health Organization International Clinical Trials Registry Platform (http://apps.who.int/trialsearch/) and the United States National Institutes of Health Ongoing Trials Register ClinicalTrials.gov (www.clinicaltrials.gov). We searched for references among the guidelines on the management of ARDS^22–24^ and the extracted articles. There were no language restrictions on any of the searches.

### Study selection and data extraction

Titles and abstracts were assessed for potential relevance independently by two reviewers (YF, YK, SO, SW, SS, NS, and RY). We retrieved the full text of study reports or publications. Two assessors independently screened the full texts, identified studies for inclusion, and verified the reasons for the exclusion of ineligible studies. Differences were resolved by discussion and, where this failed, through arbitration by a third author (CN). We contacted the authors of these studies if necessary. We recorded the selection process with appropriate details to construct a PRISMA flow diagram^18^. Data extraction was carried out using standard data extraction forms by two authors independently. Differences in opinion regarding data collection were resolved using the same methods.

### Type of outcome measures

The primary outcome was 28-day mortality. If 28-day mortality was not reported, then we used the mortality of the nearest 28 days at follow-up. The secondary outcomes were longest follow-up mortality, health-related quality of life (QOL), PaO_2_/FiO_2_ (P/F) ratio on day 1, ventilation-free day (VFD) up to 28 days, hospital length of stay (LOS), and barotrauma. Definitions of these outcomes are described in additional files (Additional File 1).

### Assessment of risk of bias

Two review authors independently evaluated the risk of bias in the included studies using the Cochrane risk of bias assessment tool, version 1^25^. These reviewers graded each potential source of bias as “high,” “low,” or “unclear.” Disagreements between the two reviewers regarding the risk of bias were resolved by discussion, with the involvement of a third reviewer (CN).

### Analysis and results synthesis

We calculated measures of treatment effects using the Cochrane Statistical Package Review Manager 5 (Cochrane Collaboration, London, UK) for data synthesis and analysis. We analyzed dichotomous data (mortality, barotrauma) as RR with 95% CIs, and continuous data (such as QOL, VFD, LOS) as mean difference (MD) with 95% CI.

We planned to perform meta-analyses for separate comparisons because of the heterogeneity of the interventions. We pooled the following predefined comparisons:

1. Comparison of target tidal volumes 4–8 ml/kg predicted body weight (PBW) or ideal body weight (IBW) and above 8 ml/kg/PBW or IBW
2. Comparison of any (author-defined) LTV and normal or HTV strategies. To identify all RCTs that compared LTV and HTV, we did not specifically target tidal volume.
3. Comparison of very low tidal ventilation (less than 6 mL/kg PBW or IBW) and low tidal ventilation (6–8 mL/kg PBW).

Studies on extracorporeal membrane oxygenation (ECMO) were qualitatively integrated and reported separately.

We used a random-effects model for data synthesis because we assumed that clinical and methodological diversity exists and that intervention effects across studies are not entirely identical. We calculated the Chi^2^ test and the I^2^ statistic to measure heterogeneity. A P-value of less than 0.1 was considered statistically significant in the Chi^2^ test. To assess publication bias, we created a funnel plot and examined Egger’s test (P < 0.05, significant reporting bias) if each comparison included more than ten studies^26,27^.

### Subgroup-analysis and sensitivity analysis

We planned subgroup analyses for primary outcomes to determine whether the results differed by one of the following: ARDS definition (the Berlin definition or not), open lung therapy (LTV plus higher positive end-expiratory pressure (PEEP) vs. HTV plus low PEEP), severity (P/F ≤ 200 mmHg or not), and control group target tidal volume (> 8 ml/kg vs 6–8 ml/kg). In sensitivity analyses, we included only studies with “low risk of bias” to assess the robustness of our conclusions for the primary outcomes. We performed another sensitivity analysis that compared LTV vs. HTV excluding studies where the average tidal volume was > 11 ml/kg on day 1 in the control group.

### Post-hoc analysis

We conducted a post hoc meta-analysis of trials conducted since 2010 comparing any very low tidal volumes (author-defined) with any LTV (author-defined). In this analysis, we included trials that assessed the usefulness of LTV during ECMO for quantitative analysis.

### Assessment of the certainty of evidence

We used the Grading of Recommendations Assessment, Development, and Evaluation (GRADE) considerations (risk of bias, consistency of effect, imprecision, indirectness, and publication bias) to evaluate the quality of the evidence based on the studies that contributed data to the meta-analyses for mortality and QOL, classifying the quality as “high,” “moderate,” “low,” or “very low”^28^. We used GRADEpro GDT software.

## Results

### Study selection

**Figure 1:**
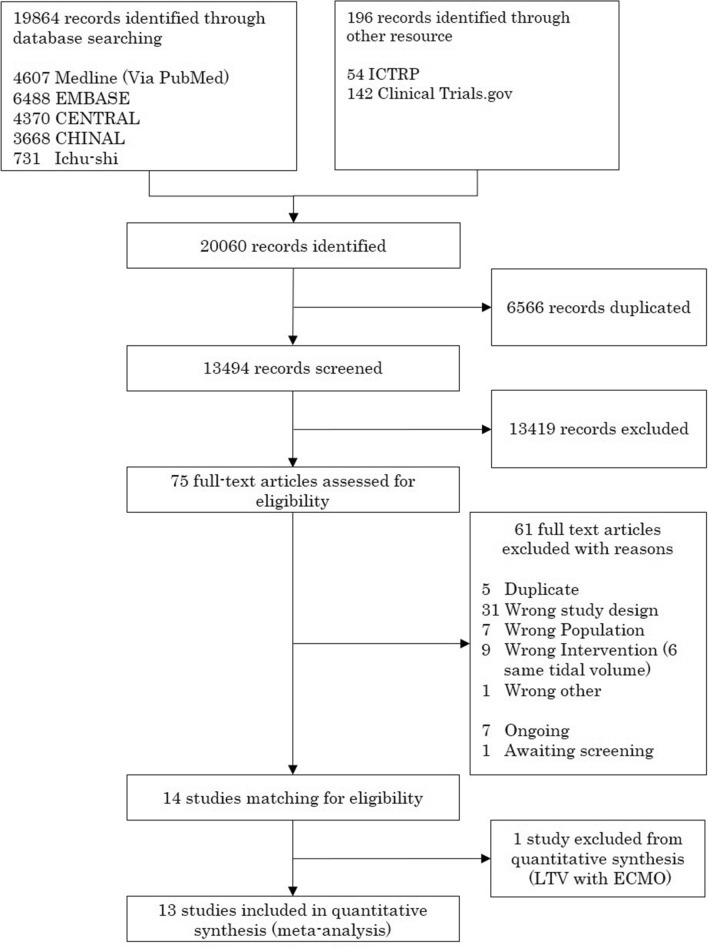
PRISMA flow diagram.

We identified 20,060 records through literature search. After removing duplicates and title and abstract screening, 75 studies were evaluated in detail and 61 were excluded (Fig. 1, Additional File 1; Supplementary Table 2). Fourteen RCTs met the eligibility criteria, and one trial assessed the usefulness of LTV during ECMO^6,11–16,29–35^. Therefore, 13 randomized trials that included 1874 patients were included in the quantitative synthesis for comparing LTV versus HTV.

### Study characteristics

The study characteristics are summarized in Table 1. For comparing LTV (4–8 ml/kg) versus HTV (> 8 ml/kg), we included 11 trials^6,11–16,30–33^. We added two trials comparing the small difference in targeting tidal volume for meta-analysis in comparison to any LTV versus any HTV^34,35^. Because one trial compared very low tidal volume ventilation with low tidal ventilation, we could not perform a meta-analysis on this comparison^34^. Tidal volumes at days 1, 3, and 7 are described in additional files (Supplementary Table 1).

**Table 1:** Characteristics of the included trials.

Study	No. of center	Definition of ARDS/ALI	*Severity P/F ratio	Intervention	Control	Mortality Outcome
**Comparison of lower tidal volume (6–8 ml/kg) versus higher tidal volume(> 8 ml/kg)**						
Amato 1998^11^	2	LISS	NA	Vt 6 ml/kg	Vt 12 ml/kg	ICU, 28-day, in-hospital
				PEEP: preset at 2 cm of water above Pflex	PEEP: stepwise algorithm for PEEP increments	
				PCV		
Brochard 1998^12^	25	LISS	NA	Vt 6–10 ml/kg PBW, Pplat < 25 cm H_2_O	Vt 10–15 ml/kg PBW, peak airway pressure < 60 cm H_2_O	30-day (from KM), 60-day
				PEEP: increments of 5 cm H_2_O (from 0 to 15) during pure oxygen breathing to determine the optimal level	PEEP: increments of 5 cm H_2_O (from 0 to 15) during pure oxygen breathing to determine the optimal level	
				VCV A/C	VCV A/C	
Stewart 1998^15^	8	Other author's definition	< 250	Vt 8 ml/kg IBW, peak pressure < 30 cm H_2_O	Vt 10–15 ml/kg IBW, peak pressure < 50 cm of water	30-day (from KM), in-hospital
				PEEP: the range of 5 to 20 cm H_2_O was adjusted in increments of 2.5 cm H_2_O	PEEP: the range of 5 to 20 cm H_2_O was adjusted in increments of 2.5 cm H_2_O	
				VCV A/C	VCV A/C	
Wu 1998^30^	1	Other author's definition	≤ 300	VT 7–10 ml/kg actual BW	VT 10–15 ml/kg	In-hospital
				PEEP: titrated to PaO_2_ (range 3–12 cm H_2_O)	PEEP: titrated to PaO_2_ (range 3–12 cm H_2_O)	
				AC and SIMV/PS	AC and SIMV/PS	
East 1999^†^^31^	10	Other author's definition	< 200	Vt 6 ml/kg IBW (titillated by computerized decision support)	Vt < 10 mL/kg IBW	In-hospital
				PEEP: Computerized Protocol, A/C	PEEP: stepwise increments of PEEP, IMV	
Brower 1999^14^	4	AECC 1994	≤ 200	Vt 5–8 mL/kg IBW, Pplat < 30 cm H_2_O	Vt 10–12 mL/kg IBW, Pplat < 55 cm H_2_O	In-hospital
				PEEP: FiO_2_ table	PEEP: FiO_2_ table	
				VCV A/C	VCV A/C	
Ranieri 1999^6^	2	AECC 1994	< 200	Vt 5 to 8 mL/kg IBW	No target, plateau airway pressure < 35 cm H_2_O	28-day
				PEEP: The PEEP was set at 2 to 3 cm H_2_O higher than the pressure at Pflex	PEEP: PEEP trial on 100% FiO_2_ was performed using incremental (3–5 cm H_2_O) levels from 3 to 15 cm H_2_O	
				VCV	VCV, maintain PaCO2 35–40 mmHg	
ARDSnet 2000^13^	10	AECC 1994	≤ 300	Vt 6 (4–8)ml/kg PBW, Pplat < 30 cm H_2_O PEEP: FiO_2_ table VCV A/C	Vt 12 ml/kg PBW, Pplat < 50 cm H_2_O PEEP: FiO_2_ table VCV A/C	30-day (from KM), Death before a patient was discharged home
Orme 2003^32^	1	Other author's definition	≤ 150	Vt 4–8 ml/kg PBW, Pplat < 40 cm H_2_O	Vt 10–15 ml/kg PBW, Pplat < 70 cm H_2_O	In-hospital
				PEEP: Computerized rules to maintain PaO_2_ above 55	PEEP: Computerized rules to maintain PaO_2_ above 55	
Villar 2006^16^	8	AECC 1994	≤ 200	Vt 5–8 mL/kg PBW	Vt of 9–11 mL/kg PBW	ICU, in-hospital, 30-day (from KM)
				PEEP: set on day 1 at Pflex + 2 cm H_2_O	PEEP: above 5 cm H_2_O, and an FiO_2_ ensuring arterial oxygen saturation 90% and PaO_2_ of 70–100 mm Hg	
				VCV A/C	VCV A/C	
Sun 2009^33^	1	Other author's definition	≤ 200	Vt 4–6 ml/kg PBW	Vt < 12 ml/kg, Pplat < 30	28-day, in-hospital
				PEEP: ARDSnet clinical trials	PEEP: ARDSnet clinical trials	
				VCV	SIMV + PS or PS	
**Comparison of any lower tidal volume versus any higher tidal volume**						
Pereira 2020^35^	5	Berlin Definition	P/F ≤ 300	Vt 4–8 ml/kg PBW, driving pressure of 10 cm H_2_O	Vt 6 ml/kg PBW, Pplat below 30 cm H_2_O	28-day, ICU, in-hospital
				PEEP: ARDSNet low-PEEP table	PEEP: ARDSNet low-PEEP table	
				VCV or PCV	VCV or PCV	
Agarwal 2013^34^	1	AECC 1994	P/F ≤ 200	Vt 6 mL/kg PBW, Pplat < 30–35 cm H_2_O	Percentage MV (%MV)	30-day (from KM), in-hospital
				PEEP: ARDSnet protocol	PEEP: ARDSnet protocol	
				VCV	ASV	
**Comparison of lower tidal volume versus higher tidal volume during ECMO**						
Thomas 2013 ^29^	10	AECC 1994	P/F < 200	Vt 3 ml/kg/PBW, assisted by avECCO2-R	Vt 6 ml/kg/PBW	In-hospital
				PEEP: ARDSNet ‘‘high-PEEP/FiO_2_’’ table	PEEP: ARDSNet ‘‘high-PEEP/FiO_2_’’ table	

*ARDS* Acute Respiratory Distress Syndrome, *ALI* Acute Lung Injury, *P/F* PaO_2_/FiO_2_, *Int* intervention, *Cont* control, *PEEP* positive end-expiratory pressure, *Pflex* lower inflection point of a pressure–volume curve, *Pplat* plateau pressure during inspiratory pause, *Vt* volume tidal, *PBW* predicted body weight, *IBW* ideal body weight, *LISS* The Lung Injury Severity Score, *Dry BW* Actual body weight minus the estimated weight gain due to salt and water retention, *VCV* volume-controlled ventilation, *PCV* pressure-controlled ventilation, *A/C* assist control, *ASV* adaptive support ventilation, *MV* mechanical ventilation, *ICU* intensive care unit, *KM* Kaplan–Meier.

*Severity used in inclusion criteria.

^†^Data extracted from Burns et al. and a subgroup with trauma-induced ARDS by McKinley et al.

### Risk of bias assessment

**Figure 2:**
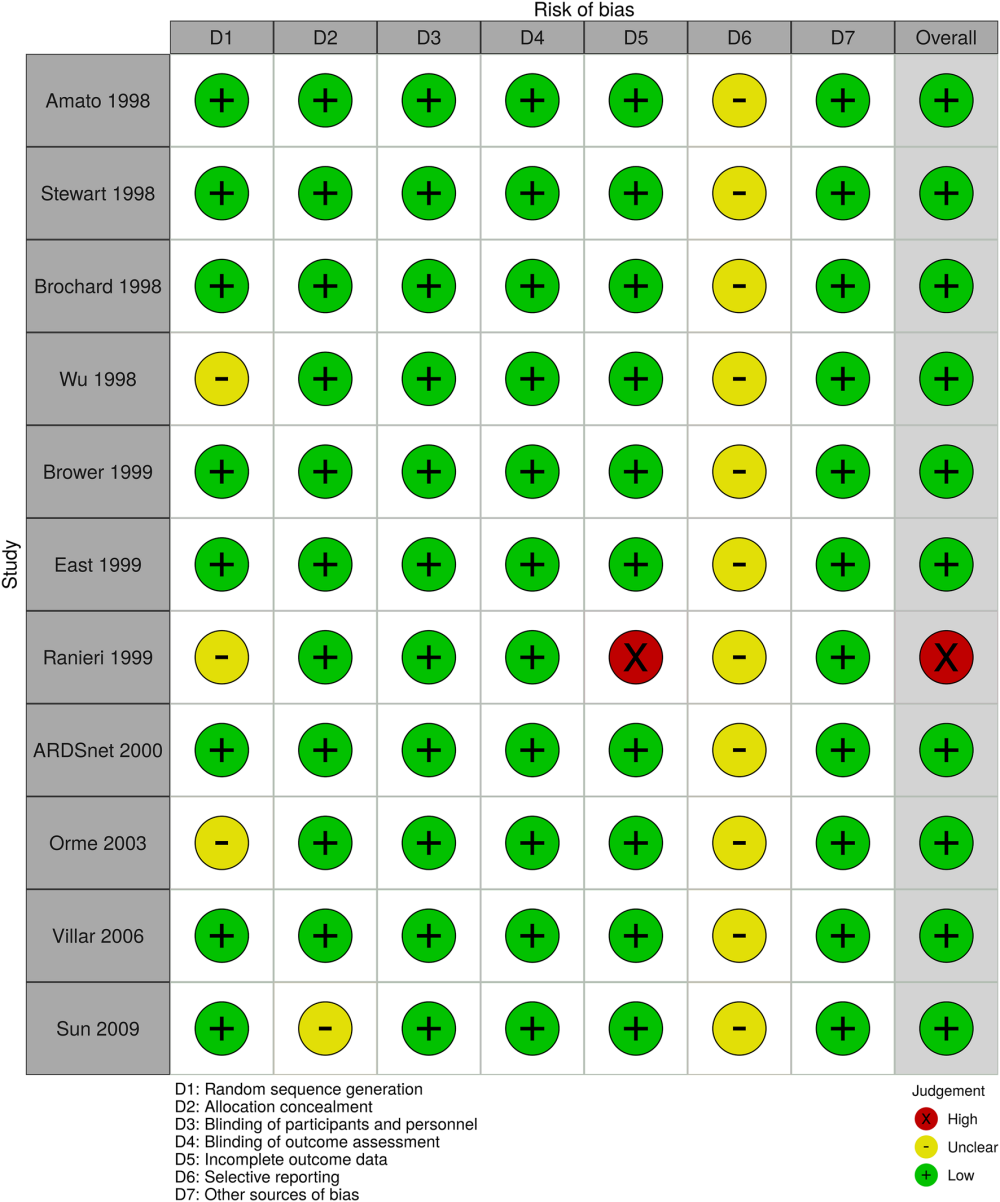
Traffic light plot of the risk of bias.

The risk of bias for mortality was low when comparing LTV (4–8 ml/kg) versus HTV (> 8 ml/kg) (Fig. 2). Masking was not performed due to the nature of the intervention in all studies, and we assessed objective outcomes such as mortality and P/F ratio as low risk of bias because it was not influenced by unmasking^36^. We evaluated subjective outcomes such as QOLs, VFD up to 28 days, LOS, and barotrauma as “unclear” risk of bias. With respect to incomplete outcomes, one study was found to have a high risk of bias because seven patients were excluded after randomization and complete-case analyses were performed for all outcomes^6^. All studies evaluated selection outcome reporting as “unclear” risk of bias because study protocols were not available. Funnel plots and Egger's test did not indicate the presence of publication bias (Egger's test p = 0.66; Supplementary Fig. 1). The risk of bias assessment of the other comparison was also similar (Additional file 1; Supplementary Figs. 2 and 3).

### Meta-analyses of the results

#### LTV (4–8 ml/kg) versus HTV (> 8 ml/kg)

**Figure 3:**
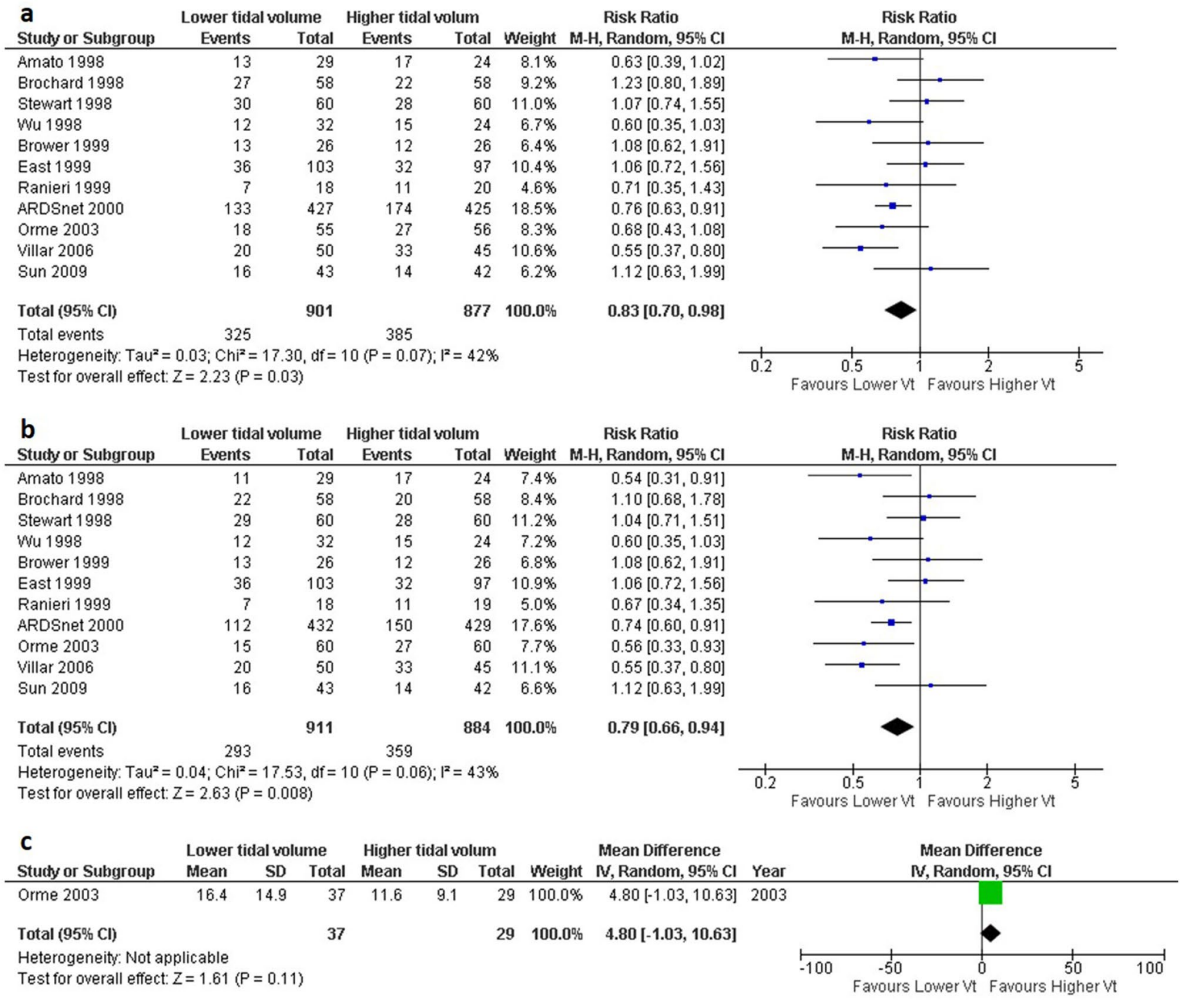
Forest plot showing the comparison of low tidal volume ventilation (LTV; 4–8 ml/kg) versus high tidal volume ventilation (HTV; > 8 ml/kg) for mortality and QOL. (**a**) 28-day mortality. Data extracted from the Kaplan–Meier curve at 28 days; Brochard 1998, Stewart 1998, ARDSnet 2000, and Villar 2006, in-hospital mortality; Wu 1998, East 1999, Brower 1999, and Orme 1999, 28-day mortality; the other studies. (**b**) the longest follow-up mortality. Data extracted from the Kaplan–Meier curve at 28 days; Villar 2006, 28-day mortality; Ranieri 1999, Sun 2009, 60-day mortality; Brochard 1998, 1-year mortality; Orme 2003. In-hospital mortality; other studies (**c**) quality of life (sickness impact profile) *CI* confidence interval; *M–H* Mantel–Haenszel method, *IV* inverse variance, *QOL* quality of life.

**Figure 4:**
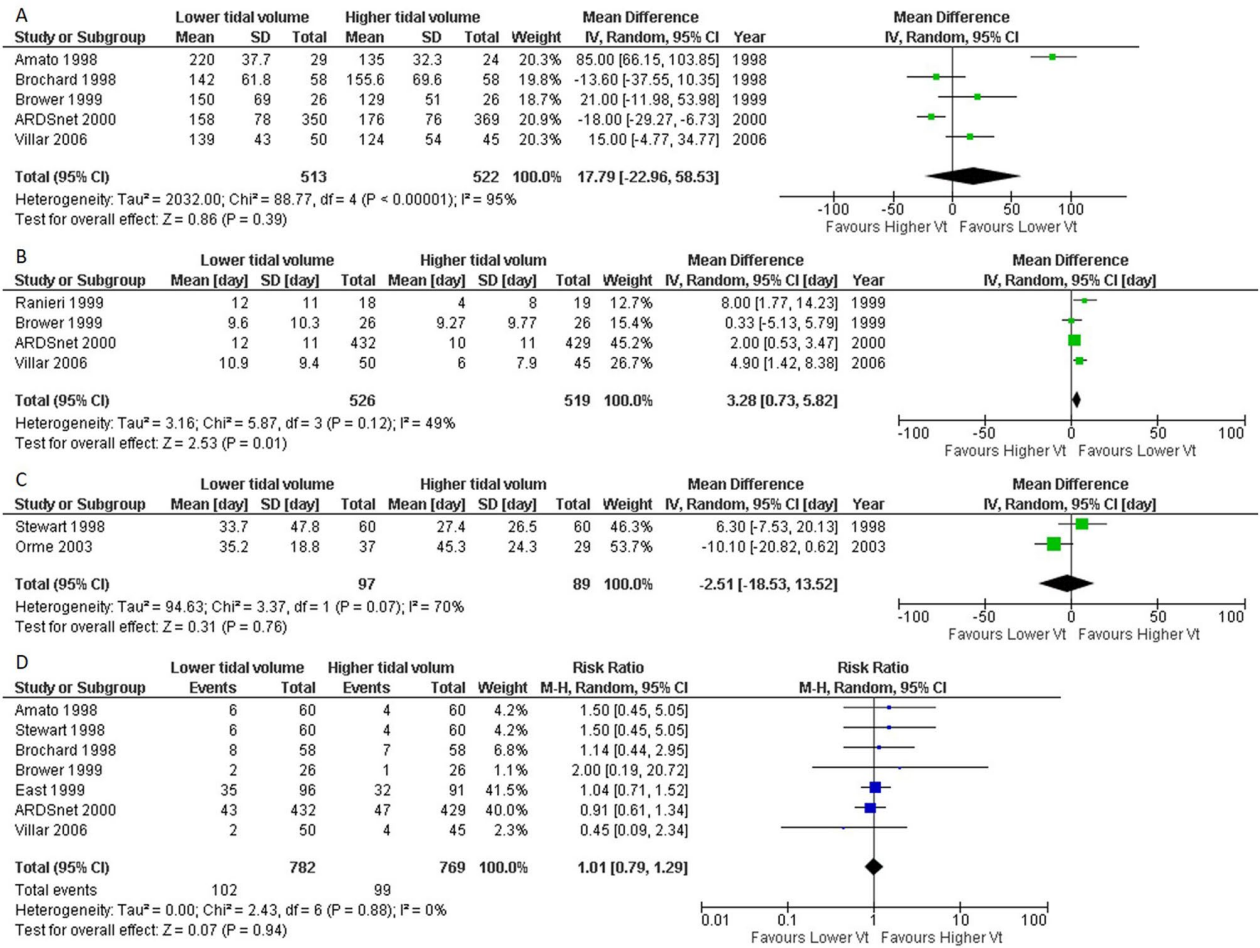
Forest plot showing the comparison of low tidal volume ventilation (LTV; 4–8 ml/kg) versus high tidal volume ventilation (HTV; > 8 ml/kg) for secondary outcomes. (**a**) PaO_2_/FiO_2_ ratio on day 1. (**b**) Ventilator-free days up to 28 days. (**c**) Length of hospital stay, (**d**) Barotrauma *CI* confidence interval, *IV* inverse variance, *M–H* Mantel–Haenszel method.

Regarding 28-day mortality and longest-follow-up mortality, the pooled RRs were · 0.79 (11 studies, 95% CI 0.66–0.94, I^2^ = 43%, n = 1795, Fig. 3A) and 0.83 (11 studies, 95% CI 0.70–0.98, I^2^ = 43%; n = 1778; Fig. 3B). Regarding QOL, only one study investigated the sickness impact profile^28^, MD was 4.80 (95% CI − 1.03–10.63, n = 66, Fig. 3C). The results of the meta-analysis for other secondary outcomes are summarized in Fig. 4. The VFD up to 28 days in the LTV group was significantly increased compared to that in the HTV group (4 studies, MD 3.28 days, 95% CI 0.73–5.82, I^2^ = 49%, n = 1045, Fig. 4B). For the other secondary outcomes, there were no significant differences between the LTV group and the HTV group (Fig. 4A, C, D).

#### Any (author-defined) LTV versus any HTV

We added two trials comparing the small difference in targeting tidal volume for the comparison of LTV versus HTV^34,35^. Actual tidal volumes at days 1, 3, and 7 are described in additional files (Supplementary Table 1). Thirteen studies were identified which evaluated the impact on mortality. The meta-analysis showed that 28-day mortality had an RR of 0.84 (13 studies, 95% CI 0.70–1.00, I_2_ = 49%, n = 1874, Supplementary Fig. 4A), and the longest follow-up mortality had an RR of 0.86 (13 studies, 95% CI 0.73–1.01, I^2^ = 45%, n = 1857, Supplementary Fig. 4B). Regarding other outcomes, there was no significant difference between any LTV and any HTV (Supplementary Fig. 4C and Supplementary Fig. 5).

### Subgroup and sensitivity analyses

Pre-planned subgroup analyses for the comparison of LTV (4–8 ml/kg) versus HTV (> 8 ml) for mortality by the definition of ARDS (Berlin vs. other), open lung therapy (LTV plus higher PEEP vs. HTV plus low PEEP), and severity of inclusion criteria (P/F ratio ≤ 200 vs. > 200) were performed. The subgroup defining ARDS could not be reported. A subgroup analysis of 11 studies reporting 28-day mortality^6,11–16,26–29^ by open lung therapy demonstrated a significant reduction in mortality (test for subgroup differences: Chi^2^ = 6.42, P for interaction 0.01; Supplementary Fig. 6A). The subgroup analysis for nine studies reporting 28-day mortality according to the severity of inclusion criteria did not show any subgroup interaction (test for subgroup differences: Chi^2^ = 0.00, P for interaction 0.98; Supplementary Fig. 6B).

In comparison to any LTV vs any HTV, subgroup analysis for 13 studies reporting 28-day mortality according to the control group target tidal volume (> 8 ml/kg vs 6–8 ml/kg) showed a significant subgroup interaction (test for subgroup differences: Chi^2^ = 0.05, P for interaction 0.02; Supplementary Fig. 7).

We performed the sensitivity analysis exploring the impact of influence of high risk of bias on the comparison of LTV (4–8 ml/kg) versus HTV (> 6 ml/kg) and found similar results (Supplementary Fig. 8). To explore the impact of influence of tidal volume of control groups, we performed another sensitivity analysis that compared LTV vs. HTV excluding studies where average tidal volume was > 11 ml/kg on day 1 in the control group. Regarding 28-day mortality and longest-follow-up mortality, the pooled RRs were 0.84 (3 studies, 95% CI 0.54–1.33, I2 = 73%, n = 331; Supplementary Fig. 9A) and 0.89 (3 studies, 95% CI 0.54–1.46, I2 = 79%; n = 331; Supplementary Fig. 9B).

### Post-hoc analysis

We performed a post hoc analysis of all trials conducted since 2010 comparing any very low tidal volumes (author-defined) with any LTV (author-defined)^29,34,35^. The analysis showed that 28-day mortality and longest-follow-up mortality were not significant, however, increase in mortality was observed in the very low tidal volume group than the LTV group (28-day mortality; RR 1.35, 95% CI 0.90–2.02, longest follow up mortality; RR 1.18, 95% CI 0.81–1.73; Supplementary Fig. 10). In addition, we described the relationship between target tidal volume and mortality in the intervention and control arms in all included studies (Supplementary Fig. 11).

### Certainty of evidence

Certainty of evidence for mortality was downgraded by one level for inconsistency and considered moderate. The certainty of evidence for QOL was low because of the serious risk of bias and had very serious imprecision (Table 2; Supplementary Table 3).

**Table 2:** Evidence tables of the systematic review. Comparison: (A) Lower tidal volume (6–8 ml/kg) compared to higher tidal volume (> 8 ml/kg) in patients with ARDS; (B) Any lower tidal volume compared to any higher tidal volume in patients with ARDS.

Certainty assessment							No of patients		Effect		Certainty
No of studies	Study design	Risk of bias	Inconsistency	Indirectness	Imprecision	Other considerations	Lower tidal volume (< 6 ml/kg)	Higher tidal volume (6-8 ml/kg)	Relative (95% CI)	Absolute (95% CI)	
**Panel A**											
11	Randomized trials	Not serious	Serious^a^	Not serious	Not serious	None	293/911 (32.2%)	359/884 (40.6%)	RR 0.79 (0.66 to 0.94)	85 fewer per 1000 (from 138 to 24 fewer)	⨁⨁⨁◯ MODERATE
11	Randomized trials	Not serious	Serious^a^	Not serious	Not serious	None	325/901 (36.1%)	385/877 (43.9%)	RR 0.83 (0.70 to 0.98)	75 fewer per 1000 (from 132 to 9 fewer)	⨁⨁⨁◯ MODERATE
1	Randomized trials	Serious^b^	Not serious	Not serious	Very serious^c^	None	37	29	–	MD 4.8 higher (1.03 lower to 10.63 higher)	⨁◯◯◯ VERY LOW

Certainty assessment							No of patients		Effect		Certainty
No of studies	Study design	Risk of bias	Inconsistency	Indirectness	Imprecision	Other considerations	Any lower tidal volume	Any higher tidal volume	Relative (95% CI)	Absolute (95% CI)	
**Panel B**											
13	Randomized trials	Not serious	Serious^a^	Not serious	Serious^d^	None	317/952 (33.3%)	375/922 (40.7%)	RR 0.84 (0.70 to 1.00)	65 fewer per 1000 (from 122 to 0 fewer)	⨁⨁◯◯ LOW
13	Randomized trials	Not serious	Serious^a^	Not serious	Serious^d^	None	349/942 (37.0%)	404/915 (44.2%)	RR 0.86 (0.73 to 1.01)	62 fewer per 1000 (from 119 fewer to 4 more)	⨁⨁◯◯ LOW
1	Randomized trials	Serious^e^	Not serious	Not serious	Very serious^d,f^	None	37	29	–	MD 4.8 higher (1.03 lower to 10.63 higher)	⨁◯◯◯ VERY LOW

*CI* Confidence interval, *RR* Risk ratio, *MD* Mean difference.

^a^Different directions of effect in the study.

^b^Most of the studies have a high risk of bias.

^c^The wide confidence interval, sample size did not reach the Optimal Information Size.

^d^The wide confidence interval.

^e^Incomplete outcome data.

^f^Unreached Optimal Information Size.

## Discussion

Our systematic review and meta-analysis showed that LTV (4–8 ml/kg) reduces 28-day mortality, longest follow-up mortality, and increase in VFD up to 28 days for adult ARDS patients. There was no significant effect on P/F ratio, QOL, LOS, and barotrauma. When comparing any LTV versus any HTV, we found a similar trend towards lower mortality with any LTV (author’s definition) in ARDS. In addition, the post-2010 study used a lower tidal volume in the control group than in the pre-2010 study.

Previous systematic reviews have reported various results^5,17,37,38^. The potential reasons for the difference between our findings and past findings include studies^6,15^, searching methods, definition of LTV, and the use of a random-effects model. We included a trial that enrolled patients at risk of developing ARDS^15^. However, the inclusion criteria for the P/F ratio were below 250 mmHg in this trial; therefore, we considered this population to be ARDS. In the 2016 Japanese ARDS guidelines, only six studies were included. We searched the literature with no language restriction and searched the trial registration database and reference for guidelines, and included 13 RCTs in our systematic review.

Another significant contribution of this review is that we investigated a comparison of any LTV versus any HTV that was included in the 13 studies. The effect size tended to be smaller than that of LTV 4–8 ml/kg compared with HTV (> 8 ml/kg). This may be due to the small difference in tidal volume between the intervention and control groups^34,35^. In a meta-regression analysis, Walkey showed that the effect tended to be smaller when the difference in the ventilation rate between the two groups was smaller^17^. Recent studies have focused on limiting the tidal volume or pressure while avoiding high tidal volumes in the control group^29,34,35^.

Our study suggests with moderate certainty that limiting the tidal volume to 4–8 ml/kg is desirable in the ventilatory management of patients with ARDS. No significant increase in harm (such as increasing barotrauma or decreasing P/F ratio) was found, but the certainty of evidence was very low. However, given the low cost and simplicity of the intervention and the survival benefit, limited tidal volume might be considered routinely. This suggestion is similar to that reported in other guidelines^22–24,39^.

Previous individual meta analysis indicated that the benefit of higher PEEP in ARDS patients receiving LTV^40^. However, this study did not examine the effect modification of higher PEEP on LTV. In our subgroup analysis, we found an effect modification when combined with a higher PEEP. This result is consistent with the results of a recent network meta-analysis^41^. Sud et al. showed that LTV combined with high PEEP was more effective than HTV, although the best effective strategy was LTV combined with prone positioning. In our study, we were unable to examine the effect modification of prone positioning on LTV due to a lack of data. The Alveolar Recruitment Trial (ART), which used very high levels of PEEP to recruit the lung showed increased mortality at 28 days^42^. If a very high PEEP is used, the effect modification on LTV may be small.

A comparison between a very low tidal volume and a lower tidal volume was not synthesized because there was only one study. Therefore, we added a post hoc analysis to compare any very low tidal volume with any low tidal volume. This meta-analysis showed that very low tidal volume tended to increase mortality compared with LTV, but this was not significant. Excessive ventilation limitations can lead to harm, but more studies are needed to verify how low tidal volumes are better. Similarly, there is a lack of evidence of LTV during ECMO.

Our study had several potential limitations. First, there was clinical heterogeneity due to differences in interventions: one study used a combined intervention with a recruitment maneuver (RM)^11^, and several studies did not describe whether they used RM^6,14,15,31,32^. Therefore, there might be heterogeneity due to RM, and there was heterogeneity because we included RCTs that examined the effect of LTV combined with high PEEP. Our subgroup analysis separately showed the effect of the combination of high PEEP and similar PEEP. Second, one study failed to complete the inclusion evaluation^43^. Chen compared pressure-limited ventilation (plateau pressure < 30 mmHg) with HTV (10–15 ml/kg), which might be included in our systematic review. However, because we could not identify the tidal volume after the intervention, this study was awaiting inclusion. Finally, in this systematic review, we performed many analyses. Caution should be exercised in the interpretation of results for secondary analyses, subgroup analyses, and sensitivity analyses.

## Conclusions

This systematic review and meta-analysis demonstrated that ventilation using LTV was associated with reduced risk of mortality in patients with ARDS compared with HTV. Our study suggests with moderate certainty evidence that limiting the tidal volume to 4–8 ml/kg is desirable in the ventilatory management of patients with ARDS. More studies are needed to verify how low tidal volumes are better.

## Supplementary Information

Supplementary Information.

## Data Availability

The data and material used for this meta-analysis were obtained from the articles in our list of references.
