# Supplementary material for: Usefulness of low tidal volume ventilation strategy for patients with acute respiratory distress syndrome: a systematic review and meta-analysis
Source: Sci Rep. 2022 Jun 4;12:9331. doi: 10.1038/s41598-022-13224-y (PMC9167294; doi:10.1038/s41598-022-13224-y)
Supplement: Supplementary file 1 — Supplementary Information. [file 41598_2022_13224_MOESM1_ESM.docx]

**Title:**

**Usefulness of low tidal volume ventilation strategy for patients with acute respiratory distress syndrome: A systematic review and meta-analysis**

Ryohei Yamamoto^1,2^, Satoru Robert Okazaki^2^, Yoshihito Fujita^3^, Nozomu Seki^4^ , Yoshufumi Kokei^5^, Shusuke Sekine^6^, Soichiro Wada^7^, Yasuhiro Norisue^8^, Chihiro Narita^9^

**Affiliations:**

^1^ Department of Healthcare Epidemiology, School of Public Health in the Graduate School of Medicine, Kyoto University, Kyoto, Japan

^2^Department of Intensive Care Medicine, Kameda Medical Center, 929 Higashi-cho, Kamogawa, Chiba, Japan

^3^Department of Anesthesiology and Intensive Care Medicine, Aichi Medical University, 1-1 Karimata, Yazako, Nagakute, Japan

^4^Emergency Department, Toyama University Hospital, 2630, Sugitani, Toyama-shi, Toyama

^5^Department of Emergency Medicine Trauma and Resuscitation Center, Tokyo Metropolitan Tama Medical Center, 2-8-29, Musashidai, Fuchu, Tokyo, Japan

^6^Department of Anesthesiology, Tokyo Medical University 6-7-1 Nishishinjyuku, Shinjuku-ku, Tokyo 160-0023, Japan

^7^Department of Pediatrics, Teine Keijinkai Hospital, 1-40, Maeda, Teine-ku, Sapporo, Hokkaido, Japan

^8^Department of Emergency and Critical Care Medicine, Tokyo Bay Urayasu Ichikawa Medical Center, 3-4-32, Todaijima, Urayasu, Chiba, Japan

^9^Departmenet of Emergency Medicine, Shizuoka General Hospital, 4-27-1, Kitaando, Aoiku, Shizuoka, Japan

***Corresponding Author**:

Ryohei Yamamoto, MD.

Department of Healthcare Epidemiology, School of Public Health in the Graduate School of Medicine, Kyoto University, Kyoto, Japan

Email: ryoheiyamamoto11@gmail.com

Phone: +81 75-753-9467

Fax: +81 75-753-4644

**Details of eligibility criteria**

**Types of participants**

Inclusion Criteria

- 16 years and older
- ARDS or ALI.
- On invasive mechanical ventilation
- The definition of ARDS or ALI is one of the following.

a) The North American-European Consensus Conference on ARDS (NAECC) [1]

b) The Lung Injury Severity Score [2]

c) The Berlin definition [3]

d) Other author's definition

Exclusion criteria

- Controlled by noninvasive ventilation (NIV)

**Type of intervention**

We included studies comparing lower tidal volume ventilation strategy with higher tidal volume ventilation strategy. The following interventions were included:

1. Comparing limiting tidal volume with a target of 4-8 ml/kg PBW or IBW or higher with the target of above 8 ml/kg/PBW

2. Comparing any (author-defined) lower tidal-volume ventilation strategies with usual or higher tidal volume ventilation strategies. We did not specifically target tidal volume. We compared any lower tidal volume target with any higher tidal volume target.

3. Comparting very low tidal volume ventilation (less than 6 ml/kg PBW or IBW) vs. low tidal volume ventilation (6-8 ml/kg PBW or IBW)

We allowed to include a variety of settings of tidal volume. For example, if there was a difference in the tidal volume between the two groups after 24-72 hours from Intervention due to differences in the method of setting of tidal volume (specifying target tidal volume, a setting of the driving pressure, any protocol, or programmatic algorithms), we will include them in the study.

Common interventions, such as the setting of the ventilation mode (VCV or PCV), different respiratory frequency settings, different PEEP methods, and recruitment maneuvers are acceptable and can be incorporated. Studies comparing lower and higher tidal volumes during ECMO were included but did not be qualitatively integrated and were reported separately. High-frequency oscillatory ventilation (HFOV) and airway pressure release ventilation (APRV) were excluded. This was because the effects of these ventilation settings were highly heterogeneous in examining differences in the effects of low tidal volume ventilation. Besides, studies in which there was no difference in actual tidal volumes after intervention were excluded, even if the targets tidal volumes are different in both groups.

**Type of outcome measures**

**Primary outcomes**

- 28-day mortality: If 28-day mortality was not reported, hospital mortality or mortality at the nearest follow-up were employed. If the Kaplan-Meier curve was reported, it was read from that figure.

**Secondary outcomes**

- Long-term mortality: longest follow-up regardless of the duration of follow-up was employed
- ADLs/QoL: A QoL measured by one of the following

a) Short Form 36 (SF-36) [4]

b) Health Assessment Questionnaire [5]

c) Sickness impact profile (SIP) [6)

d) other

- PaO_2_/FoO_2_ ratio: P/F ratio for Day 1 (not including the baseline)

- VFD28: Ventilation-free day up to 28 days.

- Barotrauma: New pneumothorax, mediastinal emphysema, subcutaneous emphysema, new thoracic drainage, or author-defined Barotrauma/Ventilation Induced Lung Injuries(VILI).

**Supplementary Table 1:** **Tidal volume at day1, 3, and 7**

| **Study** | **Tidal volume (ml/kg)** | | | | | | |
| --- | --- | --- | --- | --- | --- | --- | --- |
|  |  | **Day 1** | | **Day 3** | | **Day 7** | |
|  | **Scale** | **Int** | **Cont** | **Int** | **Cont** | **Int** | **Cont** |
| Amato 1998 | mean (SE) | 362 (11) | 763 (26) | 348 (6) | 768 (13) | NA | NA |
| Brochard 1998 | mean (SD) | 7.1 (1.3) | 10.3 (1.7) | NA | NA | 7.37 (1.3) | 10.7 (1.8) |
| Stewart 1998 | mean (SD) | 7.0 (0.7) | 10.7 (1.4) | 7.2 (0.8) | 10.8 (1.0) | 4-8 (0.6) | 10.1 (1.4) |
| Wu 1998 | NA | NA | NA | NA | NA | NA | NA |
| East 1999^†^ | NA | 9.0 | 11.0 | 8.0 | 12.0 | 8.0 | 11.0 |
| Brower 1999 | mean (SE) | NA | NA | 7.3 (0.1) ^†^ | 10.2 (0.1) ^†^ | NA | NA |
| Ranieri 1999 | mean (SD) | 7.6 (1.1) | 11.1 (1.9) | NA | NA | NA | NA |
| ARDSnet 2000 | mean (SD) | 6.2 (0.9) | 11.8 (0.8) | 6.2 (1.1) | 11.8 (0.8) | 6.5 (1.4) | 11.4 (1.4) |
| Orme 2003 | mean (SD) | 7.7 (0.84) | 11.0 (1.0) | NA | NA | NA | NA |
| Villar 2006 | mean (SD) | 7.3 (0.9) | 10.2 (1.2) | 7.1 (0.9) | 10.0 (1.0) | NA | NA |
| Sun 2009 | NA | NA | NA | NA | NA | 6.1 | 9.8 |
| Pereira 2020 | mean (SD) | 4.3 (0.5) | 5.8 (0.5) | 4.5 (0.6) | 5.7 (0.6) | 5.3 (1.2) | 5.6 (0.5) |
| Agarwal 2013 | mean | 5.7 | 6.5 | 5.7 | 6.4 | 5.8 | 6.3 |
| Thomas 2013 | mean (SD) | 5.9 (1.2) | 6.0 (0.6) | 4.2 | 6.5 | 7.1 | 8.1 |
| In Amato 1998, units of tidal volume were described as ml.  * Data extracted from Burns et a (7) l and a subgroup with trauma-induced ARDS by McKinley et al (8). † At day5 ‡ Average of 1st week SE: standard error, SD: standard deviation, NA: no data, Int: intervention, Cont: control | | | | | | | |

**Supplementary Table 2: Excluded studies**

| Study | Reason for exclusion |
| --- | --- |
| Cheng 2005 [9] | secondary analysis |
| Eisner 2001 [10] | secondary analysis |
| O'Brien 2004 [11] | secondary analysis |
| Kregenow 2006 [12] | secondary analysis |
| Amato 1995 [13] | Interim analysis of Amato 1998 |
| Hough 2005 [14] | secondary analysis |
| Kahn 2005 [15] | secondary analysis |
| Parsons 2005 [16] | secondary analysis |
| Constantin 2019 [17] | Similar tidal volume in both group |
| Hirshberg 2018 [18] | Similar tidal volume in both group |
| Hodgson 2019 [19] | Similar tidal volume in both group |
| Chen 2004 [20] | Awaiting screening |

**Supplementary Table.3 Evidence table of the systematic review for comparison of LTV(4-8ml/kg) versus HTV (>8ml/kg)**

| **Certainty assessment** | | | | | | | **№ of patients** | | **Effect** | | **Certainty** |
| --- | --- | --- | --- | --- | --- | --- | --- | --- | --- | --- | --- |
| **№ of studies** | **Study design** | **Risk of bias** | **Inconsistency** | **Indirectness** | **Imprecision** | **Other considerations** | **Lower tidal volume (<6ml/kg)** | **higher tidal volume (6-8ml/kg)** | **Relative (95% CI)** | **Absolute (95% CI)** |  |
| 11 | Randomised trials | Not serious | Serious ^a^ | Not serious | Not serious | None | 293/911 (32.2%) | 359/884 (40.6%) | **RR 0.79** (0.66 to 0.94) | **85 fewer per 1,000** (from 138 fewer to 24 fewer) | ⨁⨁⨁◯ MODERATE |
| 11 | Randomised trials | Not serious | Serious ^a^ | Not serious | Not serious | None | 325/901 (36.1%) | 385/877 (43.9%) | **RR 0.83** (0.70 to 0.98) | **75 fewer per 1,000** (from 132 fewer to 9 fewer) | ⨁⨁⨁◯ MODERATE |
| 1 | Randomised trials | Serious ^b^ | Not serious | Not serious | Very serious ^c^ | None | 37 | 29 | - | MD **4.8 higher** (1.03 lower to 10.63 higher) | ⨁◯◯◯ VERY LOW |
| 5 | Randomised trials | Not serious | very serious ^d,e^ | Not serious | Serious ^f^ | None | 513 | 522 | - | MD **17.79 mmHg higher** (22.96 lower to 58.53 higher) | ⨁◯◯◯ VERY LOW |
| 4 | Randomised trials | Not serious | Serious ^g^ | Not serious | Not serious | None | 526 | 519 | - | MD **3.28 day higher** (0.73 higher to 5.82 higher) | ⨁⨁⨁◯ MODERATE |
| 2 | Randomised trials | Not serious | very serious ^d,e^ | Not serious | Very serious ^c^ | None | 97 | 89 | - | MD **2.51 day lower** (18.53 lower to 13.52 higher) | ⨁◯◯◯ VERY LOW |
| 7 | Randomised trials | Not serious | Serious ^a^ | Not serious | Very serious ^c^ | None | 102/782 (13.0%) | 99/769 (12.9%) | **RR 1.01** (0.79 to 1.29) | **1 more per 1,000** (from 27 fewer to 37 more) | ⨁◯◯◯ VERY LOW |

**CI:** Confidence interval; **RR:** Risk ratio; **MD:** Mean difference, a. Different directions of effect in the study, b. Most of the studies have a high risk of bias, c. The wide confidence interval, the sample size did not reach the OIS, d. Different directions of effect in the study (null, effective, adverse), e. heterogeneity (I2 statistics and significant heterogeneity test ), f. The wide confidence interval, g. Inconsistency in methods of calculation for a ventilator-free day at 28

**Supplementary Table.4 Evidence table of the systematic review for comparison of any LTV versus any HTV**

| **Certainty assessment** | | | | | | | **№ of patients** | | **Effect** | | **Certainty** |
| --- | --- | --- | --- | --- | --- | --- | --- | --- | --- | --- | --- |
| **№ of studies** | **Study design** | **Risk of bias** | **Inconsistency** | **Indirectness** | **Imprecision** | **Other considerations** | **Lower tidal volume (Author's definition)** | **higher tidal volume (Author's definition)** | **Relative (95% CI)** | **Absolute (95% CI)** |  |
| 13 | Randomised trials | Not serious | Serious ^a^ | Not serious | Serious ^b^ | None | 317/952 (33.3%) | 375/922 (40.7%) | **RR 0.84** (0.70 to 1.00) | **65 fewer per 1,000** (from 122 fewer to 0 fewer) | ⨁⨁◯◯ LOW |
| 13 | Randomised trials | Not serious | Serious ^a^ | Not serious | Serious ^b^ | None | 349/942 (37.0%) | 404/915 (44.2%) | **RR 0.86** (0.73 to 1.01) | **62 fewer per 1,000** (from 119 fewer to 4 more) | ⨁⨁◯◯ LOW |
| 1 | Randomised trials | serious ^c^ | Not serious | Not serious | very serious ^b,d^ | None | 37 | 29 | - | MD **4.8 higher** (1.03 lower to 10.63 higher) | ⨁◯◯◯ VERY LOW |
| 7 | Randomised trials | Not serious | very serious ^a,e^ | Not serious | Serious ^b^ | None | 552 | 562 | - | MD **8.54 mmHg higher** (24.48 lower to 41.56 higher) | ⨁◯◯◯ VERY LOW |
| 5 | Randomised trials | Not serious | Serious ^a^ | Not serious | Serious ^b^ | None | 542 | 534 | - | MD **2.54 day higher** (0.39 lower to 5.48 higher) | ⨁⨁◯◯ LOW |
| 4 | Randomised trials | Not serious | Serious ^a^ | Not serious | Very serious ^b,d^ | None | 138 | 127 | - | MD **1.62 day lower** (7.22 lower to 3.99 higher) | ⨁◯◯◯ VERY LOW |
| 8 | Randomised trials | Not serious | Serious ^a^ | Not serious | Very serious ^b,d^ | None | 103/798 (12.9%) | 99/784 (12.6%) | **RR 1.02** (0.79 to 1.30) | **3 more per 1,000** (from 27 fewer to 38 more) | ⨁◯◯◯ VERY LOW |

**CI:** Confidence interval; **RR:** Risk ratio; **MD:** Mean difference, a. Different directions of effect in the study, b. The wide confidence interval, c. incomplete outcome data,d. Inadequate OIS, e. high I2 statistics and significant heterogeneity test

**Supplementary Figure. 1: Funnel plot for comparison of LTV(4-8ml/kg) versus HTV (>8ml/kg)**


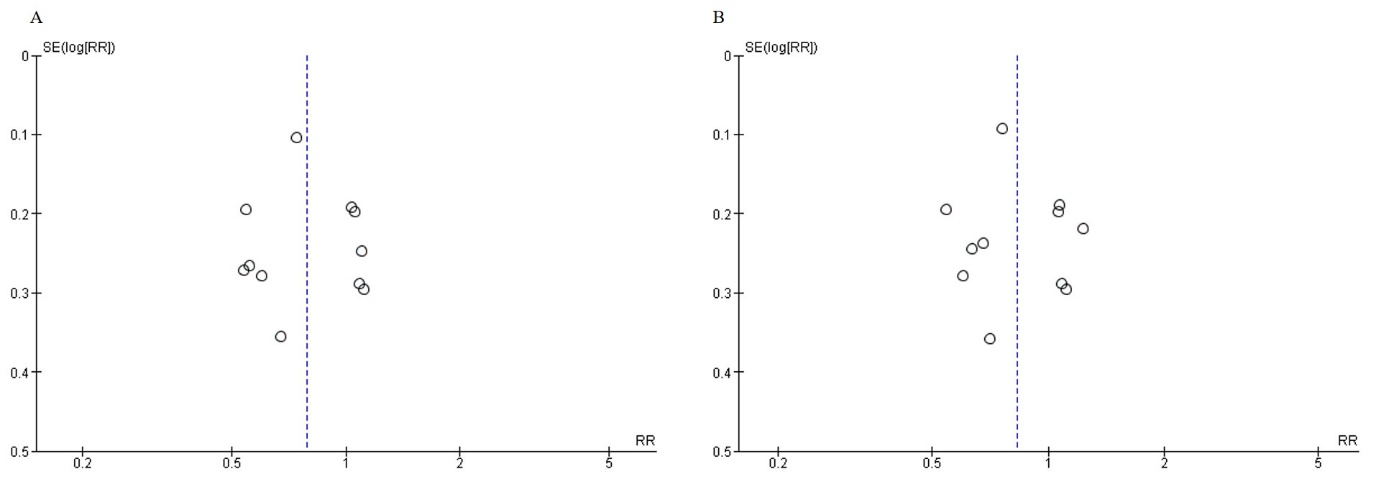


(**a**) Funnel plot of 28-day mortality, (**b**) Funnel plot of longest follow-up mortality. SE (log [RR]); standard error (log [risk ratio]), RR; risk ratio.

**Supplementary Figure. 2: Traffic light plot of risk of bias for comparison of any LTV versus any HTV**

**
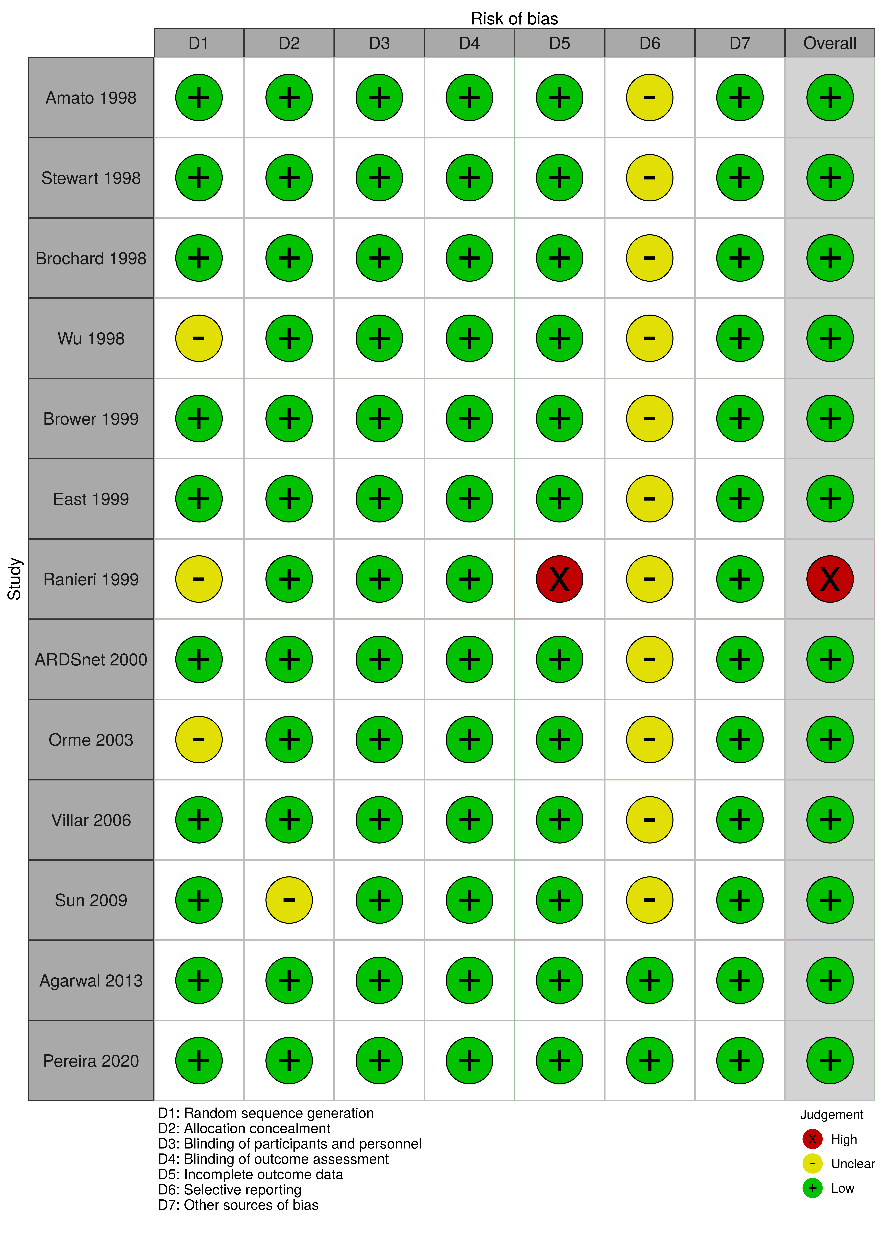
**

**Supplementary Figure. 3: Funnel plot for comparison of any LTV versus any HTV**


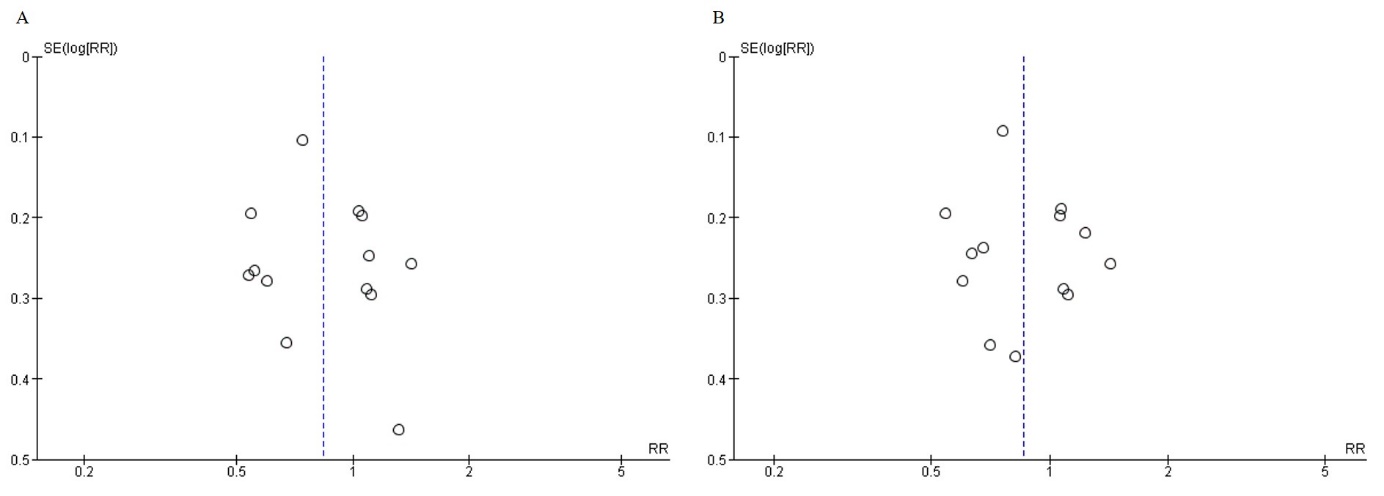


(**a**) Funnel plot of 28-day mortality, (**b**) Funnel plot of longest follow-up mortality. SE (log [RR]); standard error (log [risk ratio]), RR; risk ratio.

**Supplementary Figure. 4: Forest plot for comparison of any LTV versus any HTV for mortality and QOL**

**
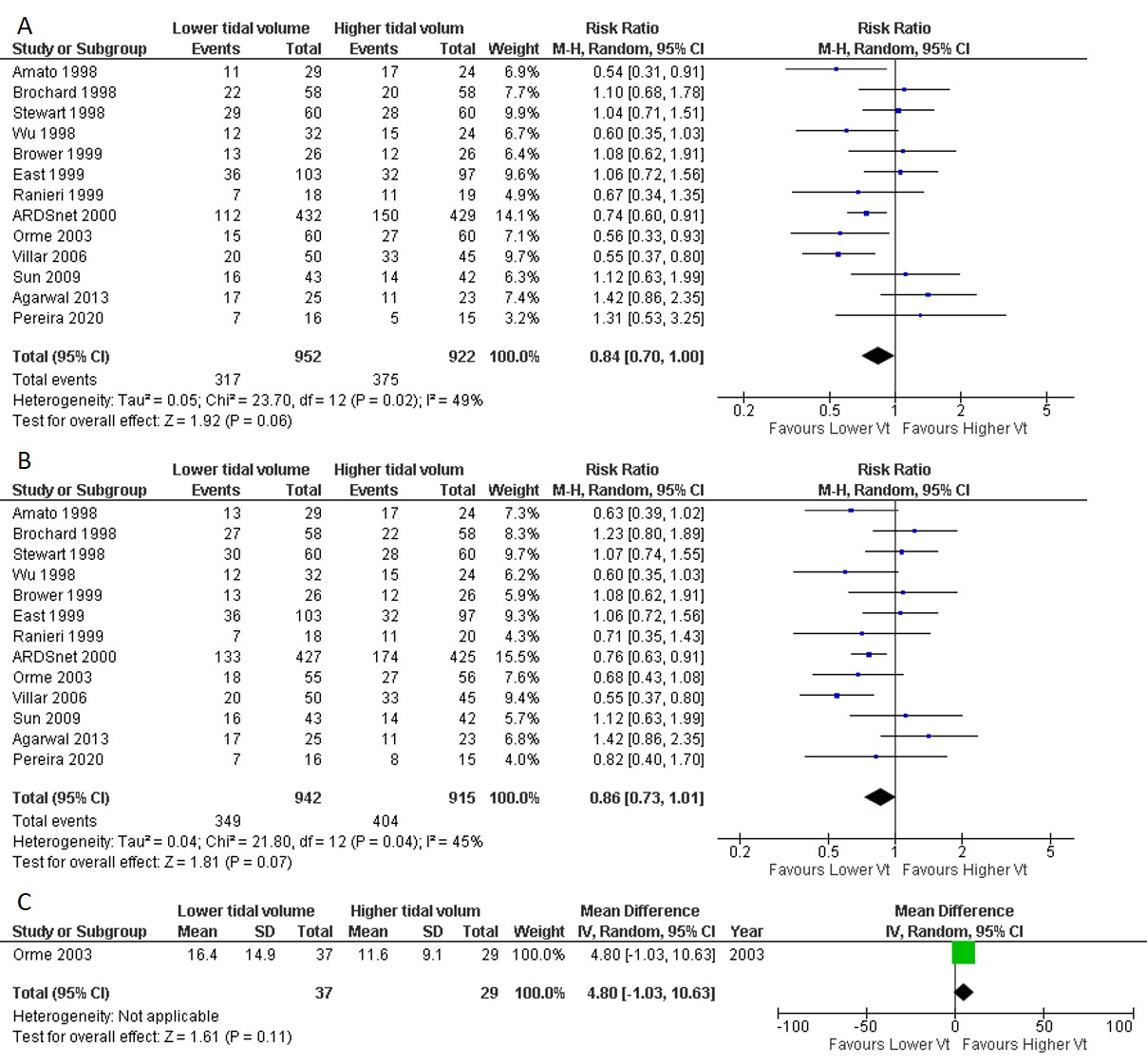
**

**(a)** 28-day mortality. Data extracted from Kaplan-Myer curve at 28 days; Brochard 1998, Stewart 1998, ARDSnet 2000, Villar 2006, and Agarwal 2013, in-hospital mortality; Wu 1998, East 1999, Brower 1999, and Orme 1999, 28-day mortality; the other studies. **(b)** the longest follower up mortality. Data extracted from Kaplan-Myer curve at 28 days; Villar 2006 and Agarwal 2013, 28-day mortality; Ranieri 1999 and Sun 2009, 60-day mortality; Brochard 1998, 1-year mortality; Orme 2003, In-hospital mortality; the other studies. **(c)** is QOL (sickness impact profile). CI: confidence interval, M–H: Mantel–Haenszel method, IV: inverse variance.

**Supplementary Figure. 5: Forest plot for comparison of any LTV versus any HTV for the secondary outcome**

**
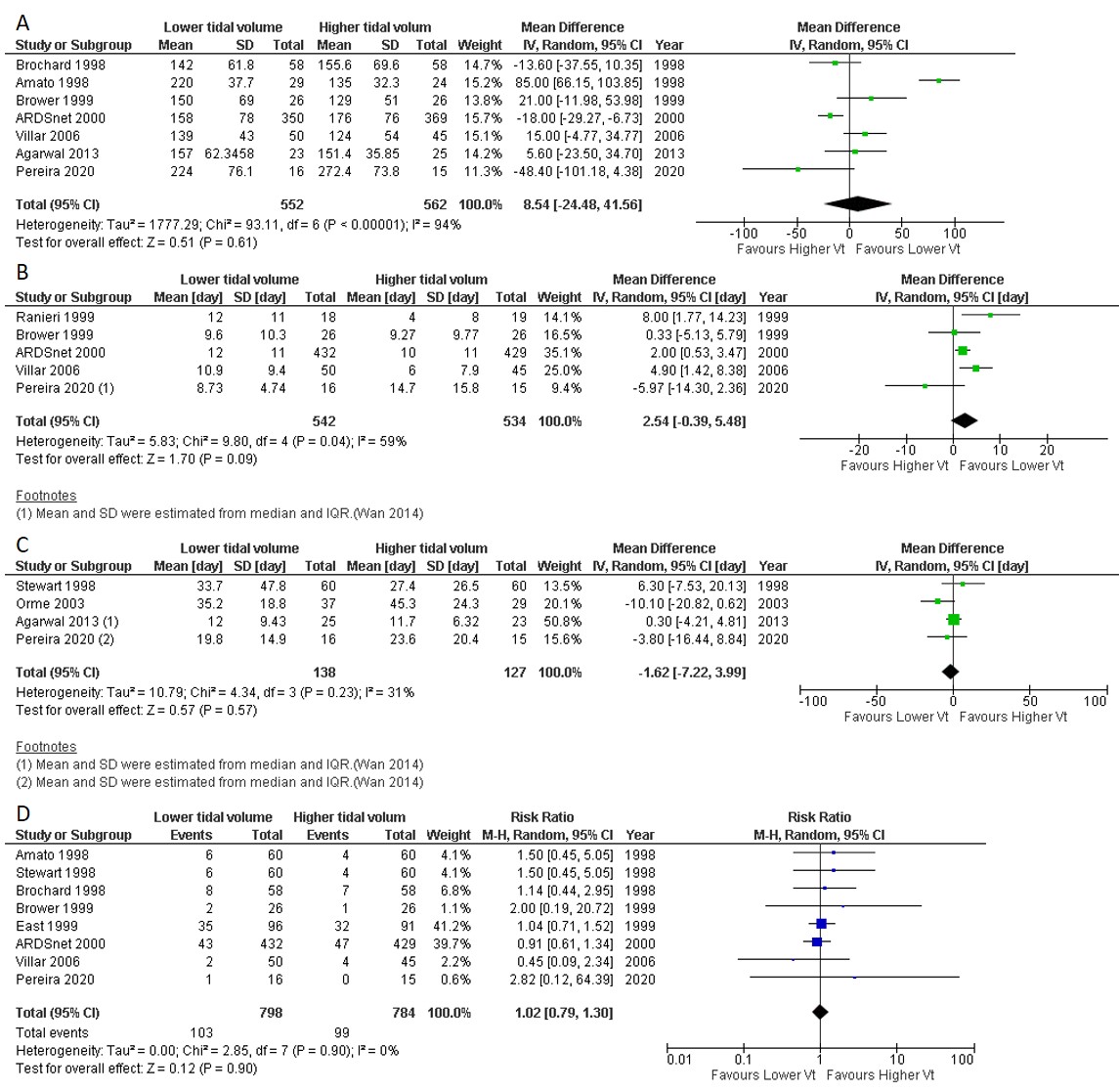
**

**(a)** PaO_2_/FiO_2_ ratio at day1. **(b)** Ventilator free day at 28. **(c)** Length of stay hospital, **(d)** Barotrauma. CI: confidence interval, IV: inverse variance, M–H: Mantel–Haenszel method.

**Supplementary Figure. 6: Forest plot of the subgroup analyses for comparison of LTV (4-8ml/kg) versus HTV (>8ml/kg)**

**
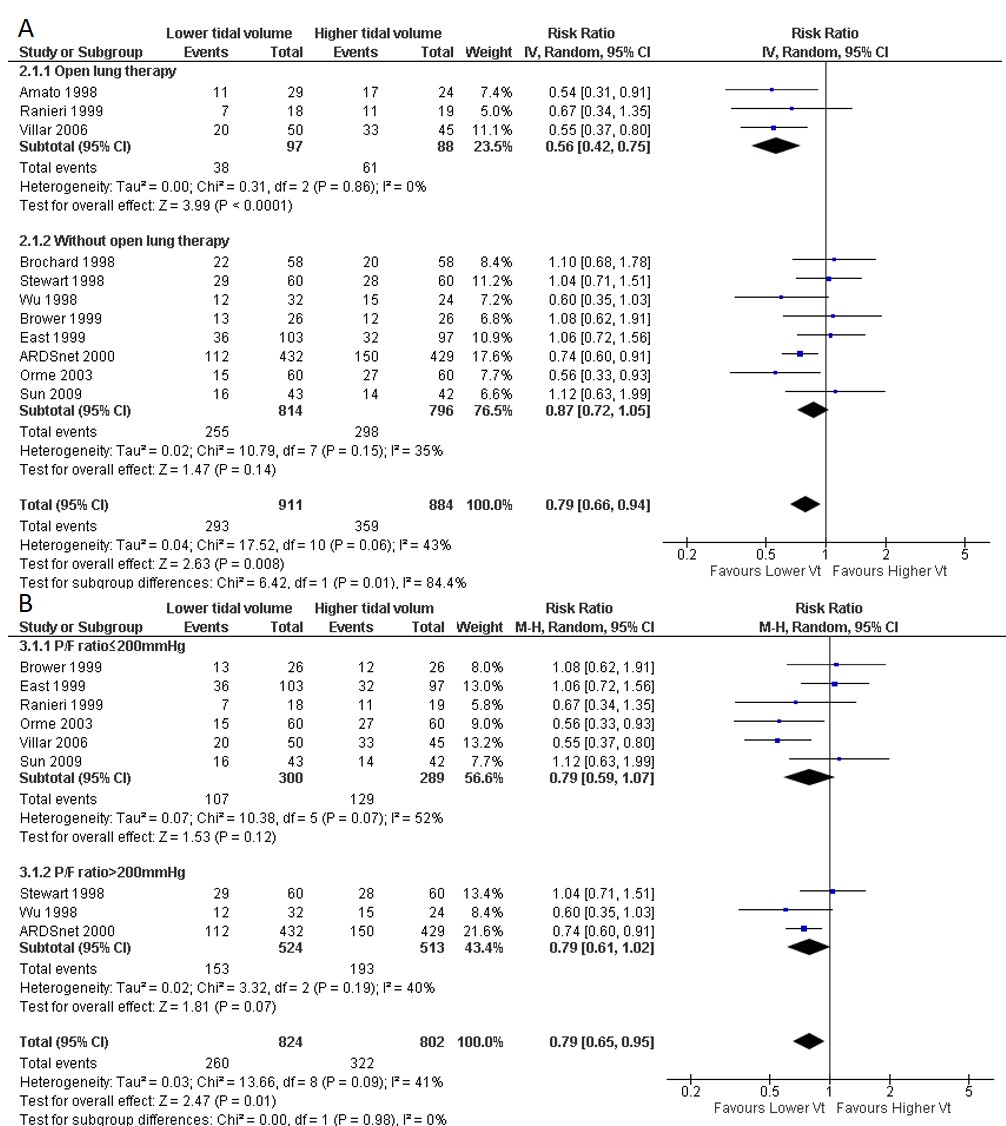
**

**(a)** 28-day mortality. **(b)** 28-day mortality.

P/F: PaO_2_/FiO_2_, CI: confidence interval, M–H: Mantel–Haenszel method.

**Supplementary Figure. 7: Forest plot of the subgroup analysis for comparison of any LTV versus any HTV (target tidal volume in the control group)
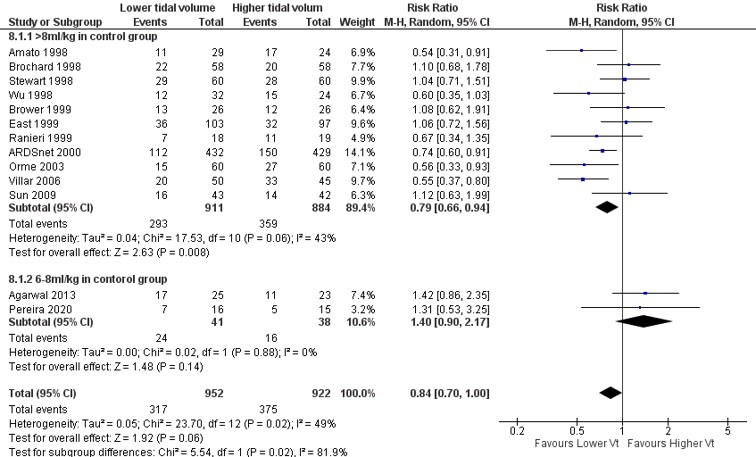
**

CI: confidence interval, M–H: Mantel–Haenszel method.

**Supplementary Figure. 8: Forest plot of the sensitivity analyses for comparison of LTV (4-8ml/kg) versus HTV (>6ml/kg) excluding the high risk of bias study**


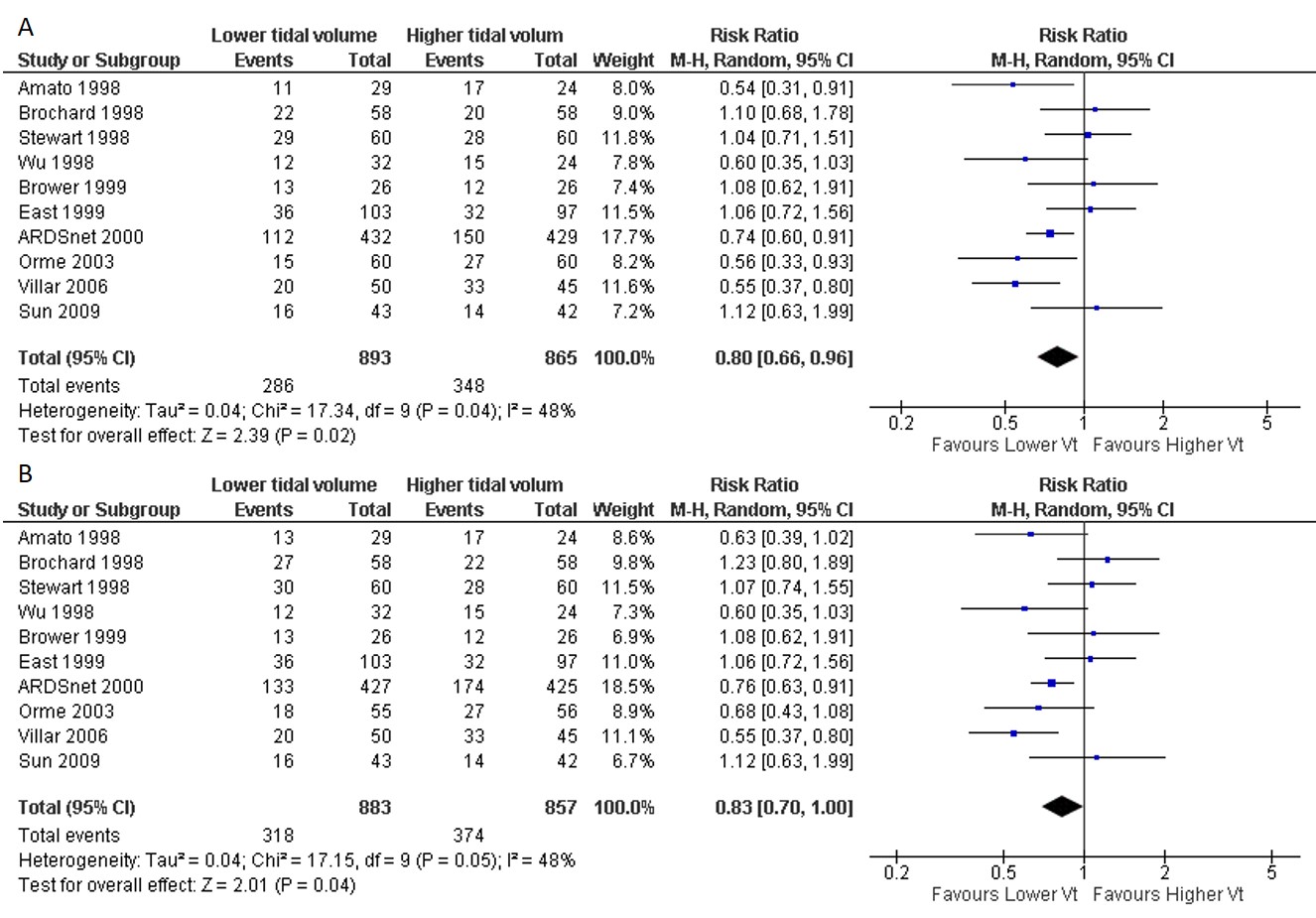


**(a)** 28-day mortality. **(b)** Longest follow-up mortality.

CI: confidence interval, M–H: Mantel–Haenszel method.

**Supplementary Figure. 9: Forest plot of the sensitivity analyses for comparison of LTV (4-8ml/kg) versus HTV (<11ml/kg).
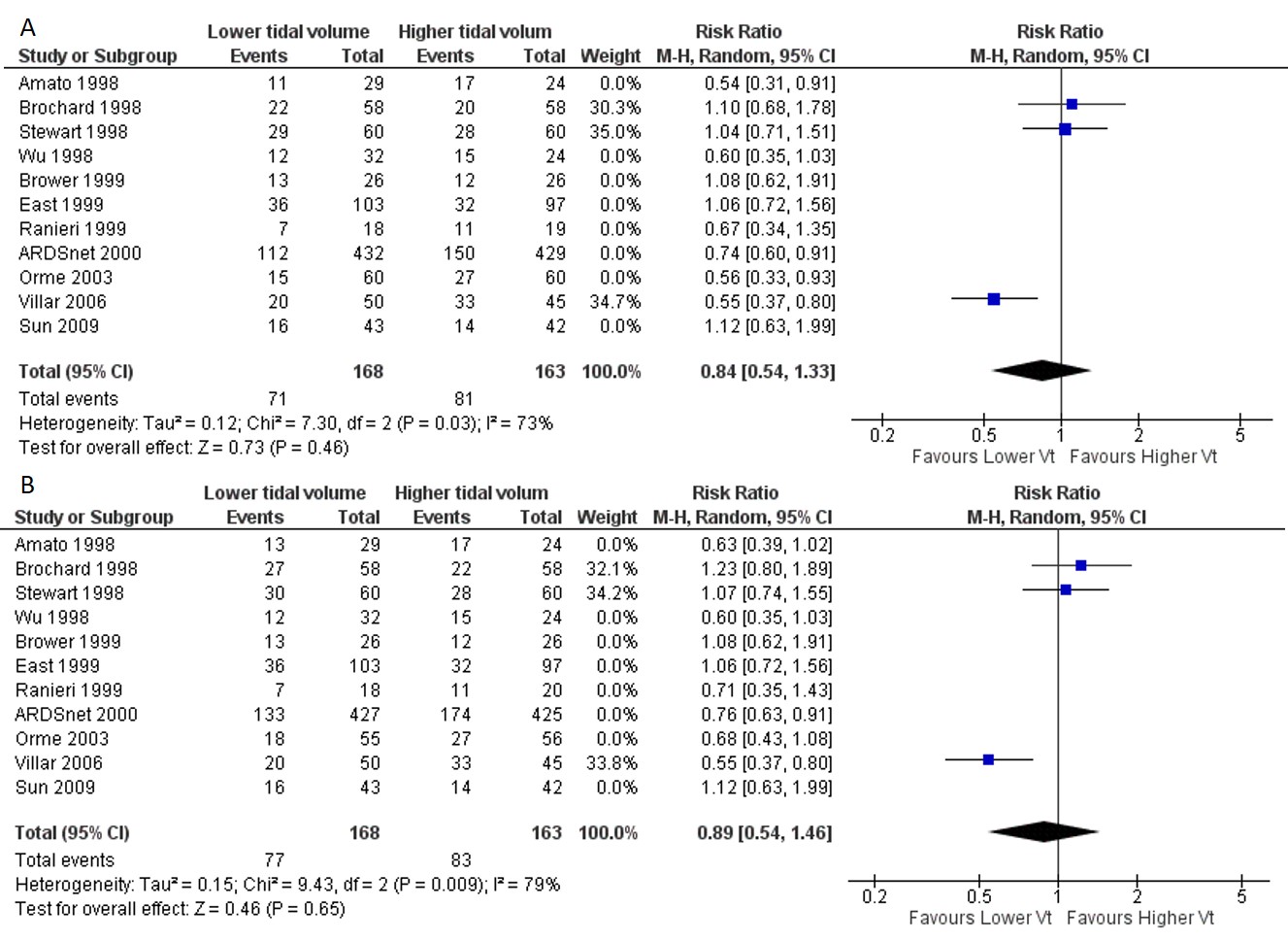
**

**(a)** 28-day mortality. **(b)** Longest follow-up mortality.

CI: confidence interval, M–H: Mantel–Haenszel method.

**Supplementary Figure. 10: Forest plot of the post hoc analysis for comparison of very low tidal volume (author-defined) versus LTV (author-defined).
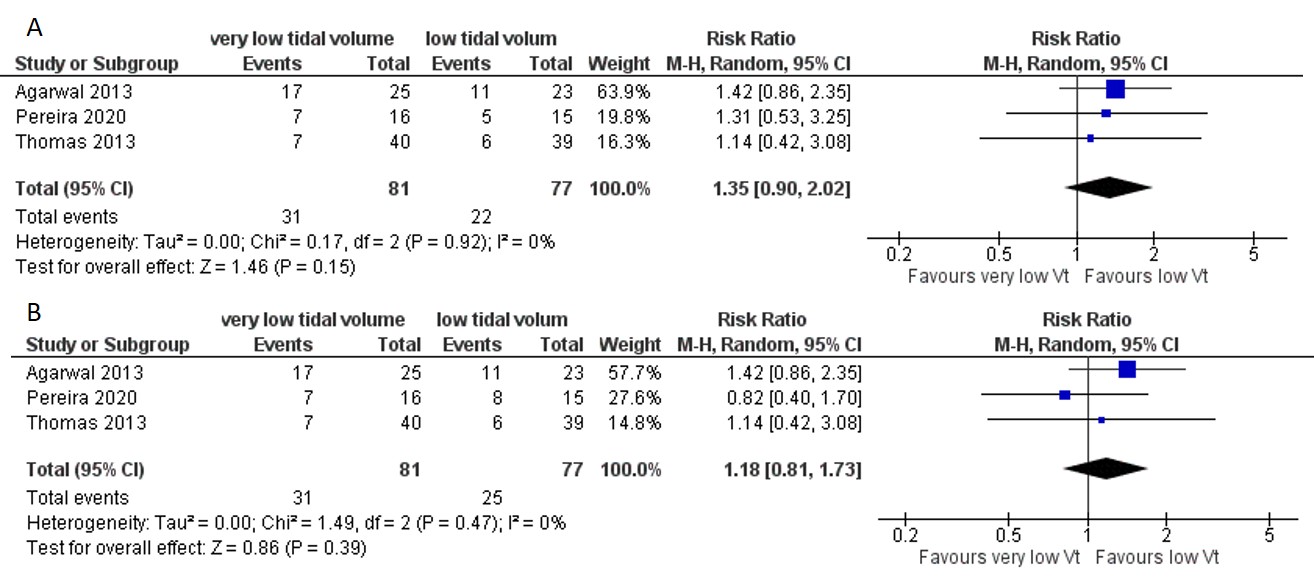
**

**(a)** 28-day mortality. In-hospital mortality; Thomas 2013, 28-day mortality; the other studies. **(b)** Longest follow-up mortality. In-hospital mortality; Thomas 2013, Pereira 2020, 28-day mortality; Agarwal 2013.

CI: confidence interval, M–H: Mantel–Haenszel method.

**Supplementary Figure. 11: Relationship between target tidal volume and mortality from both control and intervention arms.**


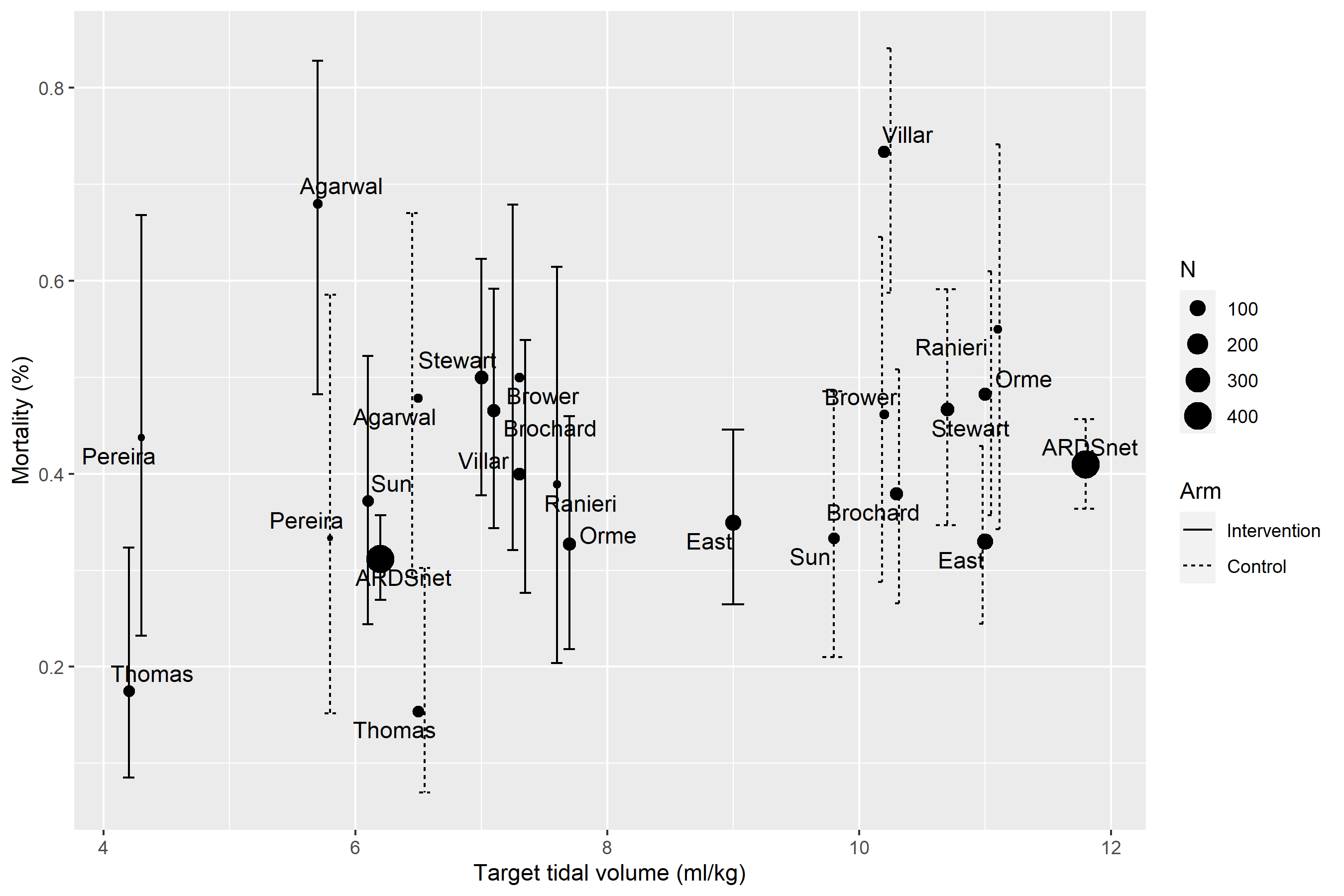


Each error bar indicates the 95% confidential interval for mortality. Target tidal volume was mean tidal volume day1 (if not available we use day 3 or 7).

**REFERENCE**

1. Artigas A, Bernard GR, Carlet J, Dreyfuss D, Gattinoni L, Hudson L, et al. The American-European Consensus Conference on ARDS, part 2. Ventilatory, pharmacologic, supportive therapy, study design strategies and issues related to recovery and remodeling. Intensive Care Med. 1998;24(4):378-98.

2. Dreyfuss D, Soler P, Basset G, Saumon G. High inflation pressure pulmonary edema. Respective effects of high airway pressure, high tidal volume, and positive end-expiratory pressure. Am Rev Respir Dis. 1988;137(5):1159-64.

3. Ranieri VM, Rubenfeld GD, Thompson BT, Ferguson ND, Caldwell E, Fan E, et al. Acute respiratory distress syndrome: the Berlin Definition. Jama. 2012;307(23):2526-33.

4. Ware JE, Jr., Kosinski M, Gandek B, Aaronson NK, Apolone G, Bech P, et al. The factor structure of the SF-36 Health Survey in 10 countries: results from the IQOLA Project. International Quality of Life Assessment. J Clin Epidemiol. 1998;51(11):1159-65.

5. Fries JF, Spitz P, Kraines RG, Holman HR. Measurement of patient outcome in arthritis. Arthritis Rheum. 1980;23(2):137-45.

6. Bergner M, Bobbitt RA, Carter WB, Gilson BS. The Sickness Impact Profile: development and final revision of a health status measure. Med Care. 1981;19(8):787-805.

7. Burns KE, Adhikari NK, Slutsky AS, Guyatt GH, Villar J, Zhang H, et al. Pressure and volume limited ventilation for the ventilatory management of patients with acute lung injury: a systematic review and meta-analysis. PLoS One. 2011;6(1):e14623.

8. McKinley BA, Moore FA, Sailors RM, Cocanour CS, Marquez A, Wright RK, et al. Computerized decision support for mechanical ventilation of trauma induced ARDS: results of a randomized clinical trial. J Trauma. 2001;50(3):415-24; discussion 25.

9. Cheng IW, Eisner MD, Thompson BT, Ware LB, Matthay MA. Acute effects of tidal volume strategy on hemodynamics, fluid balance, and sedation in acute lung injury. Crit Care Med. 2005;33(1):63-70; discussion 239-40.

10. Eisner MD, Thompson T, Hudson LD, Luce JM, Hayden D, Schoenfeld D, et al. Efficacy of low tidal volume ventilation in patients with different clinical risk factors for acute lung injury and the acute respiratory distress syndrome. Am J Respir Crit Care Med. 2001;164(2):231-6.

11. O'Brien JM, Jr., Welsh CH, Fish RH, Ancukiewicz M, Kramer AM. Excess body weight is not independently associated with outcome in mechanically ventilated patients with acute lung injury. Ann Intern Med. 2004;140(5):338-45.

12. Kregenow DA, Rubenfeld GD, Hudson LD, Swenson ER. Hypercapnic acidosis and mortality in acute lung injury. Crit Care Med. 2006;34(1):1-7.

13. Amato MB, Barbas CS, Medeiros DM, Schettino Gde P, Lorenzi Filho G, Kairalla RA, et al. Beneficial effects of the "open lung approach" with low distending pressures in acute respiratory distress syndrome. A prospective randomized study on mechanical ventilation. Am J Respir Crit Care Med. 1995;152(6 Pt 1):1835-46.

14. Hough CL, Kallet RH, Ranieri VM, Rubenfeld GD, Luce JM, Hudson LD. Intrinsic positive end-expiratory pressure in Acute Respiratory Distress Syndrome (ARDS) Network subjects. Critical Care Medicine. 2005;33(3):527-32.

15. Kahn JM, Andersson L, Karir V, Polissar NL, Neff MJ, Rubenfeld GD. Low tidal volume ventilation does not increase sedation use in patients with acute lung injury. Critical Care Medicine. 2005;33(4):766-71.

16. Parsons PE, Eisner MD, Thompson BT, Matthay MA, Ancukiewicz M, Bernard GR, et al. Lower tidal volume ventilation and plasma cytokine markers of inflammation in patients with acute lung injury. Crit Care Med. 2005;33(1):1-6; discussion 230-2.

17. Constantin JM, Jabaudon M, Lefrant JY, Jaber S, Quenot JP, Langeron O, et al. Personalised mechanical ventilation tailored to lung morphology versus low positive end-expiratory pressure for patients with acute respiratory distress syndrome in France (the LIVE study): a multicentre, single-blind, randomised controlled trial. Lancet Respir Med. 2019;7(10):870-80.

18. Hirshberg EL, Lanspa MJ, Peterson J, Carpenter L, Wilson EL, Brown SM, et al. Randomized Feasibility Trial of a Low Tidal Volume-Airway Pressure Release Ventilation Protocol Compared With Traditional Airway Pressure Release Ventilation and Volume Control Ventilation Protocols. Crit Care Med. 2018;46(12):1943-52.

19. Hodgson CL, Cooper DJ, Arabi Y, King V, Bersten A, Bihari S, et al. Maximal Recruitment Open Lung Ventilation in Acute Respiratory Distress Syndrome (PHARLAP). A Phase II, Multicenter Randomized Controlled Clinical Trial. Am J Respir Crit Care Med. 2019;200(11):1363-72.

20. Haobo C. Inspiratory plateau pressure controlling mechanical ventilation on traumatic ARDS. Chinese journal of primary medicine and pharmacy. 2004;11(2):144-5.

**Searched database**

■MEDLINE (PubMed)

■CENTRAL

■Embase

■CHINAL

■Ichu-Shi

■Other: ICTRP, Clinical.trial.gov

**MEDLINE via PubMed search strategy （until July 6, 2020）**

| 1 | Respiratory Distress Syndrome, Adult[mh] OR ARDS[tiab] OR shock lung[tiab] |
| --- | --- |
| 2 | acute respiratory distress[tiab] OR acute respiratory failure[tiab] |
| 3 | Acute[tiab] AND ((respirat*[tiab] OR ventilat*[tiab] OR pulmon*[tiab]) AND (fail*[tiab] OR depression[tiab])) |
| 4 | Lung injury[mh] OR ALI[tiab] OR Acute lung injur*[tiab] OR Ventilator-Induced Lung Injury[tiab] |
| 5 | Respiratory insufficiency[mh] OR Respiratory insufficiency[tiab] |
| 6 | Acute chest syndrome[mh] OR Acute chest syndrome[tiab] |
| 7 | #1 OR #2 OR #3 OR #4 OR #5 OR #6 |
| 8 | "Tidal volume"[mh] OR (tidal[tiab] AND volum*[tiab]) |
| 9 | ventilation AND (strateg*[tiab] OR pressure*[tiab] OR limited[tiab] OR low[tiab] OR lower[tiab] OR less[tiab] OR differen*[tiab] OR variab*[tiab] OR varying[tiab]) |
| 10 | (lung[tiab] AND protective[tiab] AND ventilat*[tiab] ) OR LPVS[tiab] |
| 11 | Respiration,artificial[mh] OR "Artificial respiration"[tiab] OR "Artificial ventilation"[tiab] |
| 12 | Ventilators, Mechanical[mh] OR "Mechanical ventilation"[tiab] |
| 13 | #8 OR #9 OR #10 OR #11 OR #12 |
| 14 | #7 AND #13 |
| 15 | ((randomized controlled trial[pt] OR controlled clinical trial[pt] OR randomized[tiab] OR placebo[tiab] OR clinical trials as topic[mesh:noexp] OR randomly[tiab] OR trial[ti] NOT (animals[mh] NOT humans [mh]))) |
| 16 | control group*[tiab] |
| 17 | #15 OR #16 |
| 18 | #14 AND #17 |

**CENTRAL search strategy （until July 6, 2020）**

| #1 | [mh "Respiratory Distress Syndrome, Adult"] OR ARDS:ti,ab OR "shock lung":ti,ab |
| --- | --- |
| #2 | "acute respiratory distress":ti,ab OR "acute respiratory failure":ti,ab |
| #3 | Acute:ti,ab AND ((respirat*:ti,ab OR ventilat*:ti,ab OR pulmon*:ti,ab) AND (fail*:ti,ab OR depression:ti,ab)) |
| #4 | [mh "Lung injury"] OR ALI:ti,ab OR "Acute lung injury":ti,ab OR " Ventilator-Induced Lung Injury":ti,ab |
| #5 | [mh "Respiratory insufficiency"] OR "Respiratory insufficiency":ti,ab |
| #6 | [mh "Acute chest syndrome"] OR "Acute chest syndrome":ti,ab |
| #7 | {OR #1-#6} |
| #8 | [mh "Tidal volume"] OR (tidal:ti,ab AND volume:ti,ab) |
| #9 | ventilation AND (strategy:ti,ab OR pressure:ti,ab OR limited:ti,ab OR low:ti,ab OR lower:ti,ab OR less:ti,ab OR different:ti,ab OR variable:ti,ab OR varying:ti,ab) |
| #10 | (lung:ti,ab AND protective:ti,ab AND ventilatory:ti,ab) OR LPVS:ti,ab |
| #11 | [mh "Respiration, Artificial"] |
| #12 | [mh "Ventilators, Mechanical"] OR "Mechanical ventilation":ti,ab |
| #13 | {OR #8-#12} |
| #14 | #7 AND #13 |
| #15 | [mh animals] NOT [mh humans] |
| #16 | #14 NOT #15 |

**Embase search strategy (until June 28, 2020)**

| S1 | (EMB.EXACT("adult respiratory distress syndrome")) OR (TI,AB(ARDS OR "shock lung")) |
| --- | --- |
| S2 | (TI,AB("acute respiratory" p/0 (distress OR failure*))) |
| S3 | (TI,AB(acute n/3 (respirat* OR ventilat* OR pulmon*) n/3 (fail* OR depression))) |
| S4 | ((EMB.EXACT("acute lung injury")) OR (EMB.EXACT("hyperoxia-induced lung injury") OR EMB.EXACT("lung injury")) OR (EMB.EXACT("ventilator induced lung injury")) OR (TI,AB(ALI OR ("acute lung" p/0 injur*) OR "ventilator-Induced lung injury"))) |
| S5 | ((EMB.EXACT.EXPLODE("respiratory failure")) OR (TI,AB(respiratory p/0 insufficien*))) |
| S6 | ((EMB.EXACT("acute chest syndrome")) OR (TI,AB("acute chest syndrome"))) |
| S7 | (S1 OR S2 OR S3 OR S4 OR S5 OR S6) |
| S8 | (EMB.EXACT("tidal volume") OR TI,AB(tidal n/2 volum*)) |
| S9 | (TI,AB(ventilation AND (strateg* OR pressure* OR limited OR low OR lower OR less OR differen* OR variab* OR varying))) |
| S10 | (TI,AB(("lung protective" n/2 ventilat*) OR LPVS)) |
| S11 | ((EMB.EXACT.EXPLODE("artificial ventilation")) OR (TI,AB(artificial p/0 (respiration* OR ventilation*)) OR (TI,AB(mechanical p/0 ventilation*)))) |
| S12 | (S8 OR S9 OR S10 OR S11) |
| S13 | (S7 AND S12) |
| S14 | ((((EMB.EXACT("controlled clinical trial") OR EMB.EXACT.EXPLODE("clinical trial (topic)") OR EMB.EXACT("randomized controlled trial")) OR (TI,AB(randomized) OR TI,AB(randomly) OR TI(trial) OR TI,AB(control p/0 group*))) NOT (ANIMAL(YES) NOT HUMAN(YES)))) |
| S15 | (S13 AND S14) |
| S16 | (S15 AND UD(>=2013)) |
| S17 | (S15 AND UD(<2013)) |

**CHINAL search strategy (until July 12, 2020)**

| #1 | (MH "Respiratory Distress Syndrome, Adult") OR TI ARDS OR AB ARDS OR TI "shock lung" OR AB "shock lung" |
| --- | --- |
| #2 | TI "acute respiratory distress" OR AB "acute respiratory distress" OR TI "acute respiratory failure" OR AB "acute respiratory failure" |
| #3 | TI Acute OR AB Acute AND ((TI respirat* OR AB respirat* OR TI ventilat* OR AB ventilat* OR TI pulmon* OR AB pulmon*) AND (TI fail* OR AB fail* OR TI depression OR AB depression)) |
| #4 | (MH "Lung injury+") OR TI ALI OR AB ALI OR TI "Acute lung injur*" OR AB "Acute lung injur*" OR TI "Ventilator-Induced Lung Injury" OR AB "Ventilator-Induced Lung Injury" |
| #5 | (MH "Respiratory Failure+")  OR TI "Respiratory Failure" OR AB "Respiratory Failure" |
| #6 | (MH "Acute chest syndrome") OR TI "Acute chest syndrome" OR AB "Acute chest syndrome" |
| #7 | S1OR S2 OR S3 OR S4 OR S5 OR S6 |
| #8 | (MH "Tidal volume") OR (TI tidal OR AB tidal AND TI volum* OR AB volum*) |
| #9 | ventilation AND (TI strateg* OR AB strateg* OR TI pressure* OR AB pressure* OR TI limited OR AB limited OR TI low OR AB low OR TI lower OR AB lower OR TI less OR AB less OR TI differen* OR AB differen* OR TI variab* OR AB variab* OR TI varying OR AB varying) |
| #10 | (TI lung OR AB lung AND TI protective OR AB protective AND TI ventilat* OR AB ventilat*) OR TI LPVS OR AB LPVS |
| #11 | (MH "Respiration, Artificial+") OR TI "Artificial respiration" OR AB "Artificial respiration" OR TI "Artificial ventilation" OR AB "Artificial ventilation" |
| #12 | (MH "Ventilators, Mechanical") OR TI “Mechanical Ventilat*” OR AB “Mechanical Ventilat*” |
| #13 | S8 OR S9 OR S10 OR S11 OR S12 |
| #14 | #7 AND #13 |
